# Genesis of ectosymbiotic features based on commensalistic syntrophy

**DOI:** 10.1038/s41598-023-47211-8

**Published:** 2024-01-16

**Authors:** Nandakishor Krishnan, Villő Csiszár, Tamás F. Móri, József Garay

**Affiliations:** 1 HUN-REN Centre for Ecological Research, Institute of Evolution, Konkoly-Thege M. Út 29-33, Budapest, 1121 Hungary; 2 Doctoral School of Biology, Institute of Biology, Eötvös Loránd University, Pázmány Péter Sétány 1/C, Budapest, 1117 Hungary; 3 Department of Probability Theory and Statistics, Eötvös Loránd University, Pázmány Péter Sétány 1/C, Budapest, 1117 Hungary; 4 HUN-REN Alfréd Rényi Institute of Mathematics, Reáltanoda U. 13-15, Budapest, 1053 Hungary

**Keywords:** Coevolution, Evolutionary theory

## Abstract

The symbiogenetic origin of eukaryotes with mitochondria is considered a major evolutionary transition. The initial interactions and conditions of symbiosis, along with the phylogenetic affinity of the host, are widely debated. Here, we focus on a possible evolutionary path toward an association of individuals of two species based on unidirectional syntrophy. With the backing of a theoretical model, we hypothesize that the first step in the evolution of such symbiosis could be the appearance of a linking structure on the symbiont’s membrane, using which it forms an ectocommensalism with its host. We consider a commensalistic model based on the syntrophy hypothesis in the framework of coevolutionary dynamics and mutant invasion into a monomorphic resident system (evolutionary substitution). We investigate the ecological and evolutionary stability of the consortium (or symbiotic merger), with vertical transmissions playing a crucial role. The impact of the ‘effectiveness of vertical transmission’ on the dynamics is also analyzed. We find that the transmission of symbionts and the additional costs incurred by the mutant determine the conditions of fixation of the consortia. Additionally, we observe that small and highly metabolically active symbionts are likely to form the consortia.

## Introduction

Examples of symbiotic interactions in which one species coexists in association with another species are frequently observed in nature and extensively studied. There is a plethora of mechanisms by which the interactions between the species form. Though definitions vary, “symbiosis” is often described as an intimate and long-term relationship of individuals of different genomes in which partners may or may not live in physical contact (often physiologically attached) and have already started to coevolve. While there is ambiguity in the definition of symbiosis and the kinds of species interactions that should fall under its purview^1^, here we adopt the above conventional definition of symbiosis^2^, which encompasses obligate symbiosis^3, 4^. The partnership can be mutually beneficial (mutualism) or asymmetrical with or without a conflict of interest (parasitism or commensalism). Ecological interactions are often complex and can be placed on a continuum varying from fitness-reducing parasitism to fitness-increasing mutualism^5, 6^. These host-symbiont associations have a long history of coevolution, and several mechanisms, in the long run, might contribute to determining the type of associations^3, 4^.

Establishing a permanent and obligate symbiosis between unicellular organisms that were once capable of independent existence is assumed to have played a crucial role in organellogenesis, eventually leading to eukaryogenesis. According to the endosymbiotic theory^7^, mitochondria and chloroplasts of eukaryotic cells descended from formerly free-living prokaryotes^8–13^. During the integration of mitochondria into an ancestor of the eukaryotic cell, a new evolutionary unit and a new level of selection emerged from free-living and independently reproducing cells, and hence is considered a major evolutionary transition^6, 14^. We develop an ecological model that describes such interactions leading to the symbiosis between free-living (not in physical contact) and unicellular individuals to provide general conditions that eventually facilitate and stabilize such interactions leading to the formation of the symbiotic entity. In other words, we are interested in the first step in the evolutionary origin of a vertically transmitted obligate symbiosis^15^. The resident ecological system that we consider is formed by two free-living species (host and symbiont), and the existence and survival of at least one species depend on the other (commensalism). This ecological dependence between these species is crucial for theoretical modeling perspectives too.

One commonly proposed ecological dependence between species is syntrophy, i.e., one species living off the metabolic products of another species^8, 9, 12, 13, 16, 17^. It is widely assumed that interactions based on syntrophy might have played a significant part in the emergence of major endosymbiotic transitions like the origin of mitochondria as an organelle^8, 9, 12, 13, 18^. Various hypotheses of eukaryogenesis also assume syntrophic interactions (hydrogen hypothesis, sulfur hypothesis, etc.)^13^ between once-independent organisms. This paper considers the initial ecological system of two species in which the host (pre-eukaryote) produces the “food” of the symbiont (ancestor of mitochondria) species. In contrast, the host is neither benefited nor harmed by the interactions. Thereby, the association can be called “syntrophic commensalism.” It is improbable that the host and symbiont, at their very first interaction and onset of their association, turned out to be immediately mutualistic with ecological compatibility. Hence, we argue that it is plausible to consider the association as a commensalistic one at the beginning, with the potential to eventually evolve into a parasitic or mutualistic one. Reciprocative metabolic exchange due to complementation (mutual or cyclic syntrophy) is assumed to be a critical factor in establishing microbial associations and forming microbial consortia, biofilms, and mats^12^. However, our model considers linear unidirectional syntrophy rather than complex mutual syntrophy. Cyclic mutual syntrophy is much more advanced, and hence, it is reasonable to assume that a relatively less sophisticated mechanism of linear and unidirectional syntrophy existed initially. For the same reason, we can assume that linear unidirectional syntrophy was more abundant, i.e., more species had this kind of relation than cyclic syntrophy. In addition, cyclic syntrophy is an example of mutualism, and our argument is that mutualism may not have evolved at the onset of interactions between two independent species. The mechanism we focus on was likely an initial step, pre-dating more complex mutualisms.

Acknowledging that the initial steps in the evolution of intimate symbiotic behavior between unicellular organisms are widely debated, we narrow our scope to a single mechanism that could set off such an association. Considering that the evolution of endosymbiosis in one step is unlikely, we hypothesize that the initial event in the evolution of physically attached intimate symbiosis between free-living organisms could be a single mutation in the symbiont genome that led to the formation of a new structure that enables it to latch onto the external surface of the host (ectosymbiosis). This single mutation is likely the first step in the evolution of intimate symbiosis. In our model, we focus on this mutated symbiont that has the potential to form an ectosymbiotic linkage with the host. The formation of this consortium benefits the symbiont because it gains more straightforward access to the substrate from the host. In this scenario, we assume that during the multiplication of the host, the ectosymbiont is not lost because it is latched onto the host’s membrane; thus, vertical transmission leads to the maintenance of the association over generations. Hence, the evolution of long-term association between the host and the symbiont species with vertical transmission is a significant concern in the evolution of eukaryotes^19^, and our model formulation tries to incorporate this.

However, microbial syntrophy cannot explain how one partner gets inside the other^13^. We suggest that the development of phagocytotic features could be a later step in the process rather than a trait that evolved before intimate symbiosis. The model can be extended to understand potential endosymbiosis, primarily from the integration of mitochondria. For instance, if the host predated the ectosymbiont by phagocytosis, that could have eventually opened the door for endosymbiosis (when the symbiont could survive within the host)^18^. In addition, adhesion to the exterior membrane might increase the likelihood of endosymbiosis. The main difference between the phagocytosis and syntrophy hypotheses is that, by phagocytosis, endosymbiosis evolved directly, while by syntrophy, ectosymbiosis evolved first. However, in both scenarios, the long-term association of the two species evolved with vertical transmission.

We introduce a coevolutionary model and are interested in whether the mutant symbiont can invade the resident ecological system. We construct a dynamical system that follows the densities of populations corresponding to our ecological model. In addition to the free-living hosts and symbionts (resident or wild type), we consider the consortia and mutant symbionts as separate “populations.” Hence, the dimension of the dynamical system is increased by two with the introduction of a single mutation, and thereby we consider a multi-species dynamical system. The successful invasion of a stable resident system by mutant phenotypes has been discussed previously^20–23^. In this paper, we are interested in the outcome of evolution by which the resident phenotype of one of the species (symbiont) is replaced by the mutant phenotype of the same species. We assume that mutation is rare enough and that multiple mutant phenotypes in the same or several species do not occur simultaneously. Evolutionary substitution occurs if the mutant phenotype can invade a stable equilibrium of the monomorphic resident system. The system evolves to a stable equilibrium of the multi-species resident-mutant coevolutionary system where the species with two phenotypes has only the mutant phenotype surviving^20, 21, 24^. Using this concept, we investigate the ecological and evolutionary stability of inter-species consortia in the framework of an ectocommensalistic association based on syntrophy. This paper aims to obtain a set of sufficient conditions that ensure the existence and local stability of the positive equilibrium point of the coevolutionary resident-mutant system with the mutant phenotype. The successful invasion and fixation of the mutant symbiont would ensure non-zero consortia density. We provide sufficient conditions for the associations to emerge and for the symbiotic merger of the host and the symbiont to stabilize in the ecological system. In essence, we consider a commensalistic system based on the syntrophy hypothesis to study a symbiogenetic model in a multi-level selection approach.

## Model

### General model for the Malthusian growth rate

Since our proposed model for ecological interactions is based on metabolic utilization and dependent on the consumption of resources by individuals of a species, it is not practical to use the conventional species growth formulations (for instance, constant growth, logistic growth, etc.). Hence, before introducing the model, we formulate a novel growth function that can capture the consumption and usage of biomass or resources along with replication. We introduce our formulation of the species’ growth rate using a generalized model of unicellular organisms that is not age structured. If the individual has collected enough biomass, i.e., when it reaches a threshold or critical quantity (cost of reproduction, $R$), it multiplies (cell division). For simplicity, cell multiplication is instantaneous, and the duration of cell division can be neglected. In other words, the duration of collecting biomass for reproduction is longer than the duration of multiplication. The time for consumption of biomass required to multiply is considered in the model, unlike usual growth models, which do not capture this. Assume that the consumption is linear, deterministic, continuous, and uniform in time with rate $L$ (i.e., the amount of biomass consumed in unit time is $L$), and the death rate of the organism is $1/P$ ($P$ is positive and $1/P$ is bounded by 1). This means an exponentially distributed lifespan with expectation $P$, i.e., the organism is alive at time $t$ with probability ${e}^{-t/P}$, supposing it has not divided by then. If the organism lives till time $R/L$, it divides. This happens with probability $\tau ={e}^{-R/PL}$. Thus, the distribution of its lifespan is exponential with parameter $1/P$ and truncated at $R/L$. This simple model of reproduction implies that the growth of the population follows a so-called Silvestrov process, which is a particular case of the general time-dependent branching processes (in other words, Crump–Mode–Jagers processes)^25^. Now we are looking for the Malthusian growth rate of the population. The expectation of the offspring size up to time $t$ is equal to $0$, if $t<R/L$ and $2\tau$, if $t\ge R/L$. This branching process is supercritical if and only if expectation of the offspring size is greater than 1 (i.e., $2\tau >1$), which implies $\frac{1}{P}<L\frac{\mathrm{ln}2}{R}$. The reproduction measure is degenerate; it puts weight $2\tau$ at $R/L$. Therefore, the Malthusian equation is $2{\tau e}^{-\alpha R/L}=1$, from which the Malthusian parameter (net growth rate) is

1 $$\alpha =\frac{L}{R}\mathrm{ln}2-\frac{1}{P} .$$

Denoting the expectation of the population size at time $t$ by ${Z}_{t}$, we have,

$$\underset{t\to \infty }{\mathrm{lim}}{e}^{-\alpha t}{Z}_{t}=\frac{R}{L\mathrm{ln}2}.$$

Moreover, the population size divided by its expectation converges almost surely to a mean one random variable, which is positive in the event of non-extinction.

### Malthusian growth rate of host species

The Malthusian growth rate of the host species depends on the following factors:

1. Consumption: We assume that the host (say species X) consumes a substrate ($\Psi$) from a nutrient source as “food.” For simplicity, we assume that the concentration of the substrate available to the host $(\psi )$ does not change in unit time (constant supply), i.e., $\Psi$ consumed by X is continuously replenished. We assume that the feeding is linear. Let ${k}_{\mathrm{X}}$ be the consumption rate of $\Psi$ by species X, then the consumption in unit time is ${k}_{\mathrm{X}}\psi .$
2. Net biomass: Let us denote the cost of living, i.e., the per capita food consumption necessary to sustain existing biomass of X, by ${C}_{\mathrm{X}}$. Thus, the net biomass available for reproduction of X during unit time is
  $${L}_{\mathrm{X}}={k}_{\mathrm{X}}\psi -{C}_{\mathrm{X}}.$$
3. Cost of reproduction: Denote ${R}_{\mathrm{X}}$ as the cost of reproduction, i.e., ${R}_{\mathrm{X}}$ is the critical quantity or threshold of biomass needed for multiplication.
4. Death rate: We assume that during the unit time, the death rate of each X individual is fixed and is denoted by ${1/P}_{\mathrm{X}}$.

Substituting all the parameters into the equation of the Malthusian parameter (Eq. (1)) for species X (host),

2 $${\alpha }_{\mathrm{X}}=\frac{{{k}_{\mathrm{X}}\psi} -{C}_{\mathrm{X}}}{{R}_{\mathrm{X}}}\mathrm{ln}2-\frac{1}{{P}_{\mathrm{X}}} .$$

Refer to Table 1 for parameter values.

**Table 1:** Variables and parameters of the model.

Variables and parameters	Meanings	Domain	Arbitrary values of parameters used for numerical analysis
$x(t)$	Density of free-living host	${\mathbb{R}}_{\ge 0}$	
$y(t)$	Density of free-living resident symbiont	${\mathbb{R}}_{\ge 0}$	
$w(t)$	Concentration of metabolic product of host species in the habitat	${\mathbb{R}}_{\ge 0}$	
$z(t)$	Density of consortia	${\mathbb{R}}_{\ge 0}$	
$u(t)$	Density of free-living mutant symbiont	${\mathbb{R}}_{\ge 0}$	
$\psi$	Concentration of nutrient source available to the host	${\mathbb{R}}_{+}$	400
$V$	Volume of the habitat or medium	${\mathbb{R}}_{+}$	10
${a}_{1}$	Competition within host species	${\mathbb{R}}_{+}$	3
${a}_{2}$	Competition within symbiont species	${\mathbb{R}}_{+}$	2
${k}_{\mathrm{X}}$	Rate of consumption of $\Psi$ by host species	(0, 1)	0.2
${k}_{\mathrm{Y}}$	Rate of consumption of W by symbiont species	(0, 1)	0.1
${C}_{\mathrm{X}}$	Cost of living of host species	(0, 5)	4
${C}_{\mathrm{Y}}$	Cost of living of resident symbiont species	(0, 2)	1.2
${C}_{\mathrm{U}}$	Cost of living of mutant symbiont species	(0, 2)	1.3
${R}_{\mathrm{X}}$	Cost of reproduction of host species	(0, 5)	3
${R}_{\mathrm{Y}}$	Cost of reproduction of resident symbiont species	(0, 1)	0.3
${R}_{\mathrm{U}}$	Cost of reproduction of mutant symbiont species	(0, 1)	0.4; 0.32
$1/{P}_{\mathrm{X}}$	Perishing or death rate of host species	(0, 1)	0.1
$1/{P}_{\mathrm{Y}}$	Perishing or death rate of symbiont species	(0, 1)	0.33
$\theta$	Effectiveness of vertical transmission	[0, 1]	1.0
$\beta$	Rate of interaction of host species and mutant symbionts	(0, 1)	0.8
$h$	Average number of ectosymbionts on the surface of a host-in-consortium	${\mathbb{R}}_{+}$	4
$\chi$	Rate of addition of free-living mutant symbionts into the habitat	${\mathbb{R}}_{+}$	30; 5

The parameter values are arbitrary, only for illustrating and verifying analytical results, and are chosen to be qualitatively consistent with biological principles.

### Malthusian growth rate of symbiont species

The Malthusian growth rate of the symbiont species depends on the following factors:

1. Consumption: W is the metabolic product of the host (species X) and is utilized by the symbionts (species Y). W gets dissolved or diluted in the surrounding habitat and is eventually utilized by individuals of species Y. For simplicity, we assume X makes exactly one molecule of W from one molecule of $$\Psi$$ it consumes. In other words, the concentration of W in the habitat ($$w$$) is proportional to the consumption of $$\Psi$$ by X. We assume the consumption of Y is linear. Let $${k}_{\mathrm{Y}}$$ be the consumption rate of W by Y in unit time. Denote by *V* the volume of the habitat. Note that $$\frac{1}{V}$$ is representative of the dilution rate of W. As the volume of the habitat increases, the concentration of dissolved W available to Y for consumption decreases. Thus, when the actual concentration is $$\frac{w}{V}$$, the net “food” consumed per time per volume by a symbiont is given by $${k}_{\mathrm{Y}}\frac{w}{V}$$. Since W dissolved in the habitat is in proportion to the consumption of food by X, the concentration $$w$$ in the habitat increases by $${k}_{\mathrm{X}}\psi x$$ in unit time (if $$x$$ is the density of species X). Indeed, all hosts in the habitat collectively consume $${k}_{\mathrm{X}}\psi x$$ molecules of $$\Psi$$. Moreover, the symbionts collectively consume $${k}_{\mathrm{Y}}\frac{w}{V}y$$ (if $$y$$ is the density of species Y), thus the concentration of W in the habitat ($$w)$$ is given by the following differential equation:
  3 $$\dot{w}={k}_{\mathrm{X}}\psi x-{k}_{\mathrm{Y}}\frac{w}{V}y .$$
  We assume that the metabolic product of X (the food for Y) is chemically stable. In other words, the degradation time of W is much longer than the reproduction time of Y; thus, Y can consume W before it is chemically degraded or transformed.
2. Net biomass: The cost of living for Y is denoted by ${C}_{\mathrm{Y}}$. Thus, the net biomass available for the reproduction of species Y in unit time is
  $${L}_{\mathrm{Y}}={k}_{\mathrm{Y}}\frac{w}{V}-{C}_{\mathrm{Y}}.$$
3. Cost of reproduction**:** Denote ${R}_{\mathrm{Y}}$ the cost of reproduction of symbiont species, i.e., ${R}_{\mathrm{Y}}$ is the biomass threshold needed for reproduction.
4. Death rate: We assume that during unit time, the death rate of each Y individual is fixed, denoted by ${1/P}_{\mathrm{Y}}$.
  Substituting all the parameters into Eq. (1), the Malthusian parameter for species Y is
  4 $${\alpha }_{\mathrm{Y}}=\frac{{{k}_{\mathrm{Y}}\frac{w}{V}}-{C}_{\mathrm{Y}}}{{R}_{\mathrm{Y}}}\mathrm{ln}2-\frac{1}{{P}_{\mathrm{Y}}} .$$

Refer to Table 1 for parameter values.

### Resident ecological system

The first step in formulating a model based on commensalistic syntrophy consists of setting up a dynamical system for the ecological dynamics of a monomorphic resident system (consisting of a single phenotype of host and symbiont species). Here we start with two free-living species in commensalistic association based on syntrophy. We assume species X (host, pre-eukaryotes) is larger (in size) than species Y (symbiont, ancestor of mitochondria), and the metabolic waste product of X is consumed by Y. The following assumptions are made to formulate the mathematical model corresponding to the resident system with free-living host and symbiont species:

1. The host acquires its “food, $\Psi$” from the environment (replenishing source). The host then produces and releases the product W to the medium as waste. Hence, there is no cost associated with the release of W. The symbionts consume W as nourishment.
2. Species Y is a commensal of species X., i.e., the symbiont is a beneficiary of the host, whereas the host is neither benefited nor harmed by the symbiont. Also, Y cannot acquire any benefit in the form of resources for its survival without the presence of X (obligate relationship). Therefore, the model based on nutrition or metabolic utilization may be called “syntrophic commensalism.”
3. No physical contact exists between resident X and Y. Thus, the concentration of the metabolic product of the host (W) available to Y for consumption is dependent on the dilution. The metabolic product W of X is diluted in the volume of the habitat or medium (*V*) where both species reside.
4. The model consists of dynamics of resource concentration of the metabolic product of host species (W), $w(t)\in {\mathbb{R}}_{\ge 0}$ and dynamics of the two populations: free-living X and free-living Y, whose densities are denoted by $x(t)\in {\mathbb{R}}_{\ge 0}$ and $y(t)\in {\mathbb{R}}_{\ge 0}$ respectively at time *t*.
5. All the consumptions are linear as given in sections "Malthusian growth rate of host species" (host) and “Malthusian growth rate of symbiont species” (symbiont).
6. In the presence of abundant resources, both population densities $x(t)$ and $y(t)$ grow at the Malthusian growth rates ${\alpha }_{\mathrm{X}}$ and ${\alpha }_{\mathrm{Y}}$ respectively as in Eqs. (2) and (4).
7. There is no interspecific competition between X and Y (for instance, for space). However, intraspecific competition bounds the densities of X and Y.
8. The concentration of W, $w(t)$ in the system depends on the densities of its producers and consumers as in Eq. (3).

Using the above assumptions, we propose the following deterministic model for the resident system using a set of non-linear (autonomous) differential equations:

$$\dot{x}=x\left(\frac{{{k}_{\mathrm{X}}\psi} -{C}_{\mathrm{X}}}{{R}_{\mathrm{X}}}\mathrm{ln}2-\frac{1}{{P}_{\mathrm{X}}}-{a}_{1}x\right)$$

$$\dot{y}=y\left(\frac{{{k}_{\mathrm{Y}}\frac{w}{V}}-{C}_{\mathrm{Y}}}{{R}_{\mathrm{Y}}}\mathrm{ln}2-\frac{1}{{P}_{\mathrm{Y}}}-{a}_{2}y\right)$$

$$\dot{w}={k}_{\mathrm{X}}\psi x-{k}_{\mathrm{Y}}\frac{w}{V}y$$

where all the parameters are strictly positive and are defined as in Table 1

### Coevolutionary system with ectosymbionts

**Figure 1:**
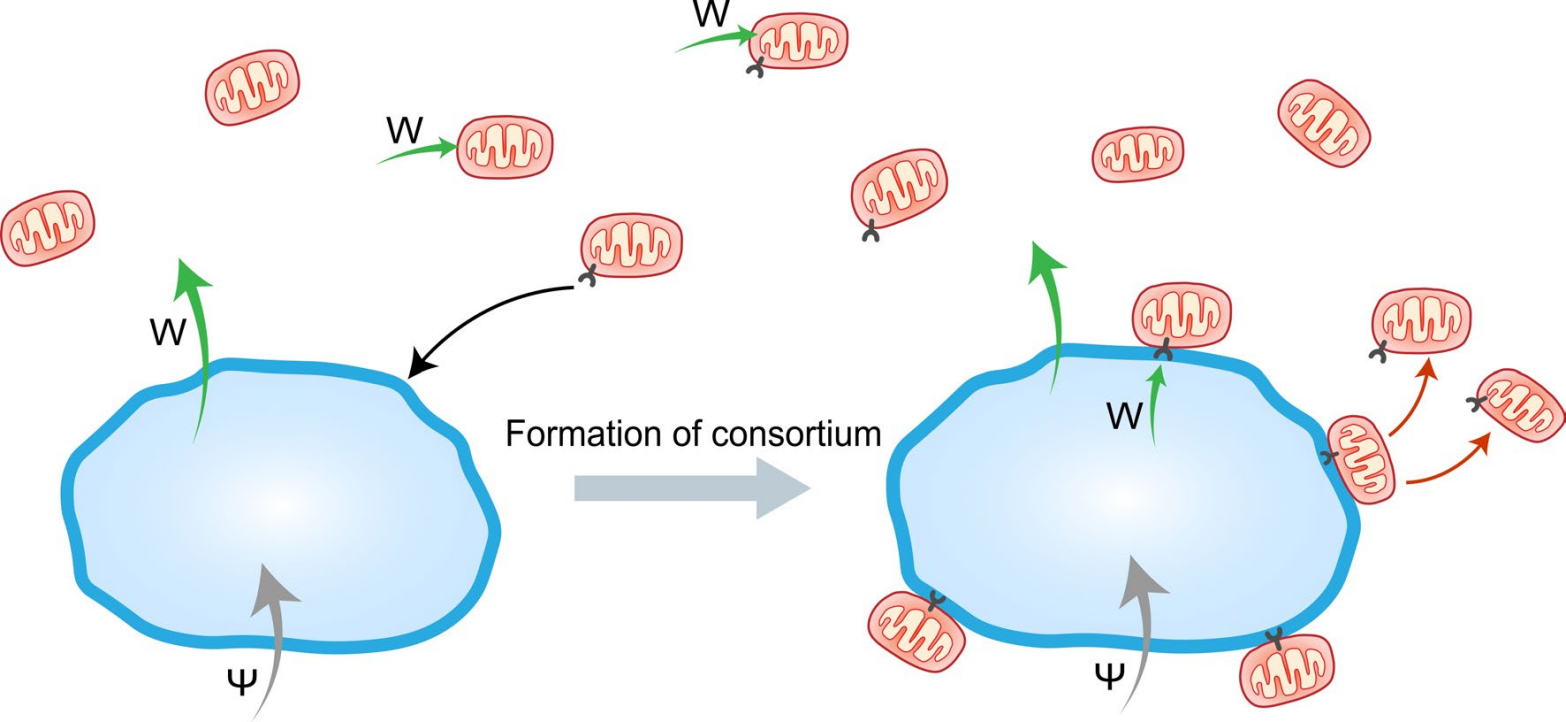
Schematic diagram of the resident-mutant coevolutionary system with host (blue) and symbiont (red) species. Grey arrows represent the flow of $\Psi$ (food of host species); green arrows represent the flow of W (metabolic product of host species); black arrow represents the formation of a physiological connection between a mutant symbiont and a host’s external surface; and red arrows represent the addition of mutant symbionts to the free-living state (due to the unsynchronized multiplication of ectosymbionts or death of host-in-consortia).

Now we consider a system (as shown in Fig. 1) that contains symbionts with a mutation in addition to the resident system. The free-living mutant symbiont U (with density $u(t)\in {\mathbb{R}}_{\ge 0}$) has a protruding structure that enables it to latch onto the external surface of its host (species X). For this, we assume that there is a membrane protein on the surface of X, to which U can connect using its extra structure. We presume that this single mutation is the first step in the formation of ectosymbiosis between the hosts and the symbionts. Due to the presence of the free-living mutant symbionts in the system, we now also have a population comprising of hosts (X) in ectosymbiotic association with mutant symbionts (U). Let us denote the ectosymbiotic “consortium or alliance (Z)” density by $z(t)\in {\mathbb{R}}_{\ge 0}$. It is important to note that the single mutation results in two extra dimensions in the coevolutionary dynamics of the entire system.

The following assumptions are made to formulate the mathematical model corresponding to the coevolutionary system with both resident and mutant phenotypes of the symbiont:

1. The “consortia, Z” can multiply in the sense that the larger member in a consortium, i.e., species X, multiplies, and the ectosymbiont is vertically transmitted during the reproduction of the “consortia.” In other words, during the multiplication of host-in-consortia, the symbiont in the association is not lost, and both parent and offspring cells of X have ectosymbionts on their surface. An offspring of a consortium has the same number of ectosymbionts as its parent. We assume perfect vertical transmission in the model for simplicity in analytical studies. However, the impact of the ‘effectiveness of vertical transmission’ is later analyzed numerically in Section "Discussion, future directions, and biological examples".
2. One free-living host can collect and accommodate more than one mutant symbiont at a time. Let us denote the average number of symbionts on one host Z as $h$. The parameter $h$ also indicates the size difference between a host and a symbiont, as a single host can associate with multiple ectosymbionts. There is a maximum threshold on the number of ectosymbionts that a host can accommodate on its surface at a time.
3. There is no competition between hosts and symbionts. However, there is competition between free-living hosts and consortia. Also, there is competition between free-living resident symbionts and free-living mutant symbionts.
4. We assume that the ectosymbionts do not alter any characteristics of the host. We make the following assumptions on the characteristics of the free-living hosts and hosts-in-consortia.
  - We assume that the presence of ectosymbionts does not inhibit the feeding of the hosts (i.e., diffusion of $\Psi$ into the host and the consumption rate of the substrate $\Psi$ by X remains the same, i.e., ${k}_{\mathrm{X}}={k}_{\mathrm{Z}}$.
  - The death rate of X remains the same i.e., ${P}_{\mathrm{X}}={P}_{\mathrm{Z}}$.
  - The cost of living of X remains the same, i.e., ${C}_{\mathrm{X}}={C}_{\mathrm{Z}}$.
  - The cost of reproduction of X remains the same, i.e., ${R}_{\mathrm{X}}={R}_{\mathrm{Z}}$.
  Observe that above assumptions (a)–(d) imply that ${\alpha }_{\mathrm{X}}={\alpha }_{\mathrm{Z}}$. All these suggest that our model is an example of commensalism wherein the host in the alliance gets neither benefit nor loss. We also do not expect the host to mount an immune response as the host has no negative effect from the presence of symbionts. Thus, as an immune response would have no advantage, a costly immune response would not be adaptive.
5. The free-living mutant symbionts consume the dissolved W, much like the free-living resident symbionts. The consumption rate of dissolved W by U is the same as that of Y, i.e., ${k}_{\mathrm{Y}}={k}_{\mathrm{U}}$. Also, we assume that the connecting protein and the connection do not alter the consumption rate.
6. Once connected with a host, a mutant symbiont can consume the metabolic product W directly from its host at the rate ${k}_{\mathrm{U}}$. $h{k}_{\mathrm{U}}{k}_{\mathrm{X}}\psi$ of the metabolic products of a host-in-consortia (Z) is directly consumed by the ectosymbionts, i.e., there is no element of dilution, and hence access to the resource is better. Thus, only $(1-{k}_{\mathrm{U}}h){k}_{\mathrm{X}}\psi z$ part of the metabolic products of all Z is released into the habitat. If the ectosymbionts can consume all metabolic products W produced by a host-in-consortium, then no amount of W is dissolved into the habitat. Once connected to the host surface, the consumption of W by the ectosymbionts directly from the host is high enough that the consumption of dissolved W from the habitat is neglected.
7. Y has an extra cost due to the novel mutation (surface structure) and thereby, ${C}_{\mathrm{U}}>{C}_{\mathrm{Y}}$, where ${C}_{\mathrm{U}}$ and ${C}_{\mathrm{Y}}$ are the costs of living of U and Y, respectively
8. Similarly, Y also needs more biomass to reproduce due to the presence of the additional structure, ${R}_{\mathrm{U}}>{R}_{\mathrm{Y}}$, where ${R}_{\mathrm{U}}$ and ${R}_{\mathrm{Y}}$ are the costs of reproduction of U and Y, respectively.
9. For simplicity, we assume competition-independent death rates for free-living resident and mutant symbionts, i.e., ${P}_{\mathrm{Y}}={P}_{\mathrm{U}}$.
10. Since free-living mutant symbionts only consume dissolved metabolic product W, their Malthusian growth rate is given by,
  5 $${\alpha }_{\mathrm{U}}=\frac{{{k}_{\mathrm{U}}\frac{w}{V}}-{C}_{\mathrm{U}}}{{R}_{\mathrm{U}}}\mathrm{ln}2-\frac{1}{{P}_{\mathrm{U}}} .$$
11. The encounter of free-living U and X is random, i.e., proportional to $xu$. Random encounters integrate new alliances with probability $\beta \in (\mathrm{0,1})$.
12. We assume that the connection in a consortium is effective and that there is no dissociation. However, the addition of mutant symbionts into the medium/ habitat can occur due to two phenomena.
  - The ectosymbiont could replicate along with its host (i.e., replication of consortia) or independently if the ectosymbionts reproduce faster than the host. The latter possibility of unsynchronized multiplication of ectosymbionts releases free-living mutant symbionts into the habitat. If we assume symbionts can reproduce faster than the host and the reproduction rate of the ectosymbionts on the surface of the host is high enough, the Z individual produces mutant symbionts which are emitted (at a constant rate) to the habitat as free-living mutant symbionts.
  - When a host-in-consortia dies, free-living mutant symbionts are added back to the medium or habitat at a constant rate. In other words, the ectosymbionts become free-living.

We assume the total addition or inclusion of free-living mutant symbionts into the medium is at a constant rate $\chi \in {\mathbb{R}}_{+}.$ This addition also allows horizontal transmission of symbionts, i.e., transmission from consortia to free-living hosts. Note that high values of $\chi$ indicate a high possibility of horizontal transmission of mutant symbionts as it linearly increases the density of free-living mutant symbionts (in the habitat).

Based on the above assumptions, we propose the following deterministic model for the dynamics of the resident-mutant coevolutionary system by using a set of autonomous non-linear differential equations:

$$\dot{x}=x\left(\left(\frac{{{k}_{\mathrm{X}}\psi} -{C}_{\mathrm{X}}}{{R}_{\mathrm{X}}}\mathrm{ln}2-\frac{1}{{P}_{\mathrm{X}}}\right)-{a}_{1}(x+z)\right)-\frac{\beta xu}{h}$$

$$\dot{y}=y\left(\frac{{{k}_{\mathrm{Y}}\frac{w}{V}}-{C}_{\mathrm{Y}}}{{R}_{\mathrm{Y}}}\mathrm{ln}2-\frac{1}{{P}_{\mathrm{Y}}}-{a}_{2}(y+u)\right)$$

$$\dot{z}=z\left(\left(\frac{{{k}_{\mathrm{X}}\psi} -{C}_{\mathrm{X}}}{{R}_{\mathrm{X}}}\mathrm{ln}2-\frac{1}{{P}_{\mathrm{X}}}\right)-{a}_{1}\left(x+z\right)\right)+\frac{\beta xu}{h}$$

$$\dot{u}=u\left(\frac{{k}_{\mathrm{Y}}\frac{w}{V}-{C}_{\mathrm{U}}}{{R}_{\mathrm{U}}}\mathrm{ln}2-\frac{1}{{P}_{\mathrm{Y}}}-{a}_{2}(y+u)\right)-\beta xu+\chi z$$

$$\dot{w}={k}_{\mathrm{X}}\psi x{+{(1-k}_{\mathrm{Y}}h)k}_{\mathrm{X}}\psi z-\frac{{k}_{\mathrm{Y}}w}{V}\left(y+u\right)$$

where all the parameters are strictly positive and are defined as in Table 1.

### Stability analysis and evolutionary substitution

From an evolutionary perspective, beneficial (adaptive) mutations are infrequent, so it is reasonable to argue that evolution is a step-by-step process. Hence, we assume only one mutation, i.e., the mutant symbionts have a novel structure (mutation) that enables them to latch onto the external surface of the host. We also assume that the ecological equilibrium sets sooner than a back mutation or any other mutation happens. We are interested in whether the mutant symbiont can invade a stable equilibrium of the monomorphic resident system and replace the resident phenotype. Evolutionary substitution of the resident phenotype by the mutant phenotype of the symbionts occurs if the system evolves to a stable equilibrium of the resident-mutant coevolutionary system where the symbiont species has only the mutant phenotype surviving. In other words, the requirements for evolutionary substitution are:

1. The resident system with no mutant phenotype is stable, i.e., there is species coexistence in the resident system. Mathematically, there exists a locally asymptotically stable interior (positive) equilibrium to the resident system.
2. The mutant phenotype of exactly one species can invade, i.e., the resident-only equilibrium of the resident-mutant system is not locally asymptotically stable.
3. The resident-mutant system must evolve to a locally asymptotically stable equilibrium with all species present, but the one species with both resident and mutant (invading) phenotypes will have only the mutant phenotype surviving. In other words, the resident phenotype of one species existing in the resident system is substituted by the invading mutant phenotype.

## Results

**Figure 2:**
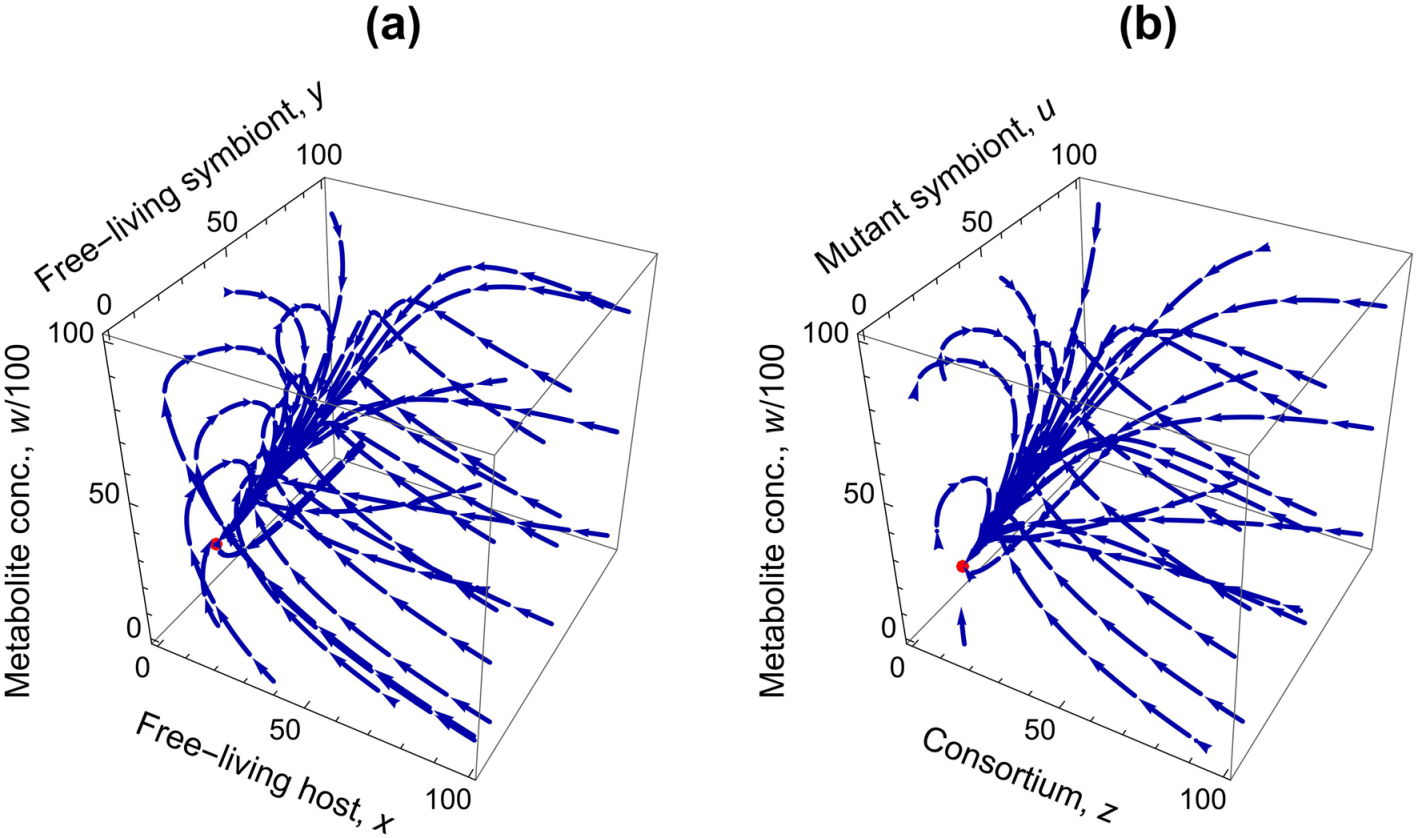
(**a**) Phase-portrait of the resident-only system (3D) with trajectories tending to ${E}_{R}({x}^{*},{y}^{*},{w}^{*})=(5.82, 22.43, 2076)$; (**b**) Phase-portrait of the mutant-only system (3D) with trajectories tending to ${E}_{M}({z}^{+},{u}^{+},{w}^{+})=(5.82, 17.51, 1595)$. The values of the parameters of the dynamics are listed in Table 1.

The monomorphic resident system is analyzed for the stability conditions of its fixed points, which is one of the primary objectives of our study. The resident system has two biologically feasible equilibrium points: the trivial equilibrium point ${E}_{0}(0, 0, 0)$ and the interior equilibrium point ${E}_{R}({x}^{*},{y}^{*},{w}^{*})$. Both equilibria exist unconditionally; ${E}_{0}(0, 0, 0)$ is unstable always and ${E}_{R}({x}^{*},{y}^{*},{w}^{*})$ is locally asymptotically stable always (see Supplementary Information, SI 1 for proof). Thus, we have unique commensalistic dynamics in which if all the parameters are positive, then we have a locally asymptotically stable interior rest point in three-dimension (Fig. 2a). In other words, the resident system is stable, and the host and the symbiont species coexist in the absence of mutant phenotypes. Also, we can observe that the mutant-only equilibrium $({E}_{M}({z}^{+},{u}^{+},{w}^{+}))$ of a mutant-only system with consortia and mutant phenotype of the symbiont always exists and is locally asymptotically stable unconditionally (see Fig. 2b; see Supplementary Information, SI 2 for details and proof).

The five-dimensional coevolutionary resident-mutant system is analyzed to find the sufficient conditions that would enable the ecological and evolutionary stability of the intimate consortium (formed by the physiological integration of the host and the symbiont species that were once capable of independent mobility), which is the significant aim of this paper. The resident-mutant system has four biologically feasible equilibria: ${E}_{0}(\mathrm{0,0},\mathrm{0,0},0)$, ${E}_{1}({x}^{*},{y}^{*},{0, 0, w}^{*})$, ${E}_{2}(0, 0, {{z}^{+}, {u}^{+}, w}^{+})$ and ${E}_{3}(0,\widetilde{y}, \widetilde{z}, \widetilde{u}, \widetilde{w})$. It is worthwhile to note that there does not exist an interior equilibrium point $(x, y, z, u, w)$ of the five-dimensional coevolutionary dynamics such that all $x,y,z,u,w\in {\mathbb{R}}_{+}$. In other words, positive-valued interior equilibria do not exist for the resident-mutant system, and there are only boundary equilibria in the system.

The trivial equilibrium point ${E}_{0}(\mathrm{0,0},\mathrm{0,0},0)$ and the resident trivial equilibrium point ${E}_{1}({x}^{*},{y}^{*},{0, 0, w}^{*})$ exist unconditionally and are always unstable (see Theorem 2.1 in Supplementary Information, SI 2 for proof). Biologically, this means that the mutant phenotype of the symbiont can invade the resident system. Additionally, for the evolutionary substitution of the resident phenotype by the mutant phenotype of the symbiont, we check whether the resident-mutant system evolves to a locally asymptotically stable equilibrium with the mutant phenotype of the symbiont.

Mathematically, the mutant equilibrium point of the resident-mutant system ${E}_{2}(\mathrm{0,0},{{z}^{+}, {u}^{+}, w}^{+})$ exists unconditionally and is locally asymptotically stable for the following conditions. In other words, the following are the sufficient conditions that guarantee the invasion and subsequent fixation of the mutant system with the intimate consortium:

1. $$\frac{{C}_{\mathrm{Y}}}{{R}_{\mathrm{Y}}}>\frac{{C}_{\mathrm{U}}}{{R}_{\mathrm{U}}}$$ and
2. $$\chi >\frac{{k}_{\mathrm{X}}\psi \left(\mathrm{ln}2\right)\left(1-{k}_{\mathrm{Y}}h\right)\left({R}_{\mathrm{U}}-{R}_{\mathrm{Y}}\right)}{{R}_{\mathrm{U}}{R}_{\mathrm{Y}}} .$$

See Theorem 2.2 in Supplementary Information, SI 2 for proof and Supplementary Information, SI 4 for verification of the non-zero probability of evolutionary substitution under arbitrary parameter values. It is important to note that our results are fully analytical and not fully numerical. Whatever parameter choices one plugs in would work if biologically consistent with our model. See Supplementary Information, SI 3 and SI 4, for better clarity on parameter choices where evolutionary substitution is assured. Our results hold for a broader range of parameters.

**Figure 3:**
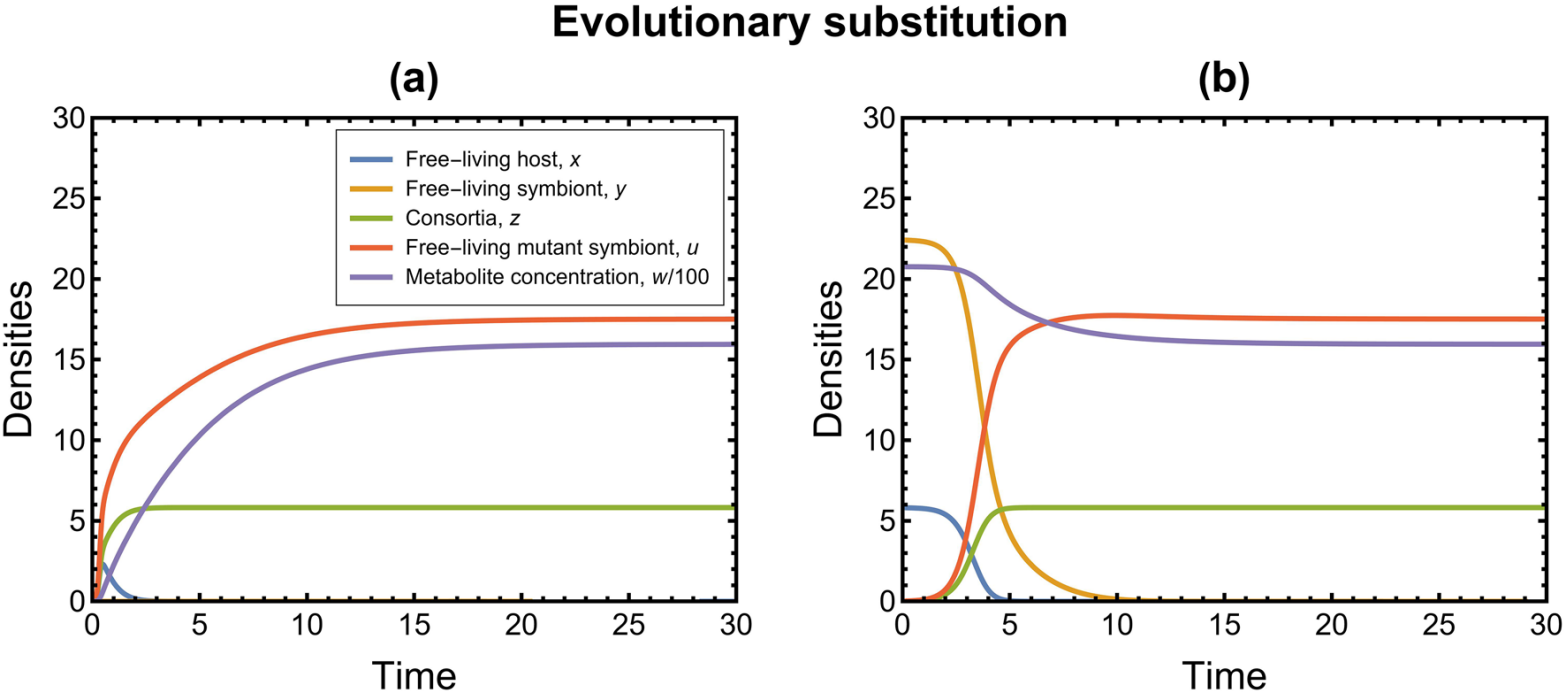
Time series plots of the resident-mutant system (5D) corresponding to evolutionary substitution showing the stability of ${E}_{2}(0, 0,{{z}^{+}, u}^{+},{w}^{+})$ when $\chi =30$ and ${R}_{\mathrm{U}}= 0.4$. Values of rest of the parameters are listed in Table 1; (**a**) For perturbed trivial equilibrium ${E}_{0}\left(0, 0, 0, 0, 0\right)$, observe that the dynamics attain stability at ${E}_{2}(0, 0,{{z}^{+}, u}^{+},{w}^{+})=(0, 0, 5.82, 17.51, 1595)$. ${E}_{0}\left(0, 0, 0, 0, 0\right)$ is unstable and ${E}_{2}(0, 0,{{z}^{+}, u}^{+}, {w}^{+})$ is stable; (**b**) For perturbed resident equilibrium ${E}_{1}({x}^{*}, {y}^{*},{0, 0, w}^{*})=(5.82, 22.43, 0, 0, 2076)$, observe that the dynamics attain stability at ${E}_{2}(0, 0,{{z}^{+}, u}^{+},{w}^{+}) =(0, 0, 5.82, 17.51, 1595)$. ${E}_{1}({x}^{*},{y}^{*},{0, 0, w}^{*})$ is unstable and ${E}_{2}(0, 0, {{z}^{+}, u}^{+},{w}^{+})$ is stable. See Supplementary Information, SI 3 for more details.

**Figure 4:**
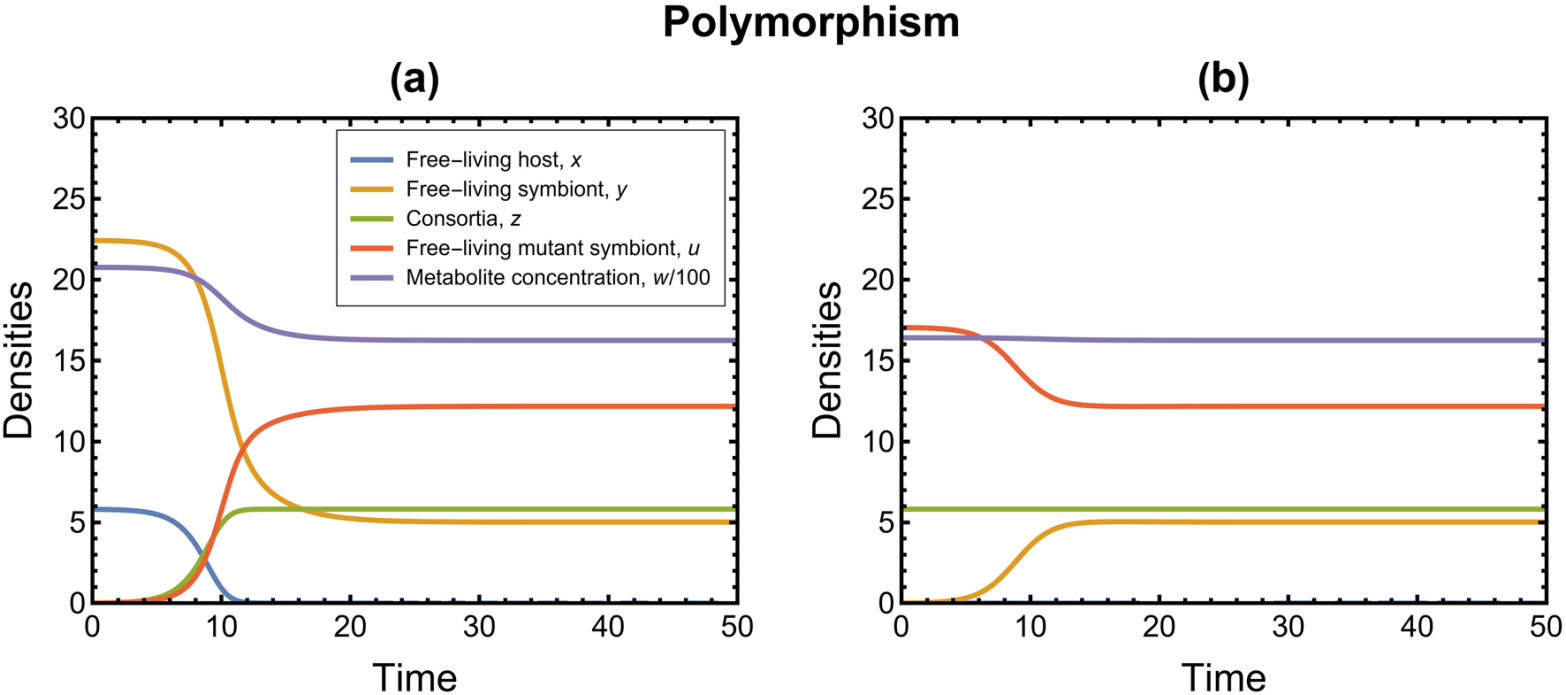
Time series plots of the resident-mutant system (5D) corresponding to polymorphism showing the stability of ${E}_{3}(0,\widetilde{y}, \widetilde{z}, \widetilde{u}, \widetilde{w})$ when $\chi =5$ and ${R}_{\mathrm{U}}= 0.32$. Values of rest of the parameters are listed in Table 1; (**a**) For perturbed resident equilibrium ${E}_{1}({x}^{*},{y}^{*}, {0, 0, w}^{*})=(5.82, 22.43, 0, 0, 2076)$, observe that the dynamics attain stability at ${E}_{3}(0,\widetilde{y}, \widetilde{z}, \widetilde{u}, \widetilde{w})=(0, 5.02, 5.82, 12.19, 1623.7)$. ${E}_{1}({x}^{*},{y}^{*},{0, 0, w}^{*})$ is unstable and ${E}_{3}(0,\widetilde{y}, \widetilde{z}, \widetilde{u}, \widetilde{w})$ is stable; (**b**) For perturbed mutant equilibrium ${E}_{2}(0, 0,{{z}^{+}, u}^{+},{w}^{+})=(0, 0, 5.82, 17.04, 1639.7)$, observe that the dynamics attain stability at ${E}_{3}(0,\widetilde{y}, \widetilde{z}, \widetilde{u}, \widetilde{w})=(0, 5.02, 5.82, 12.19, 1623.7)$. ${E}_{2}(0, 0,{{z}^{+}, u}^{+},{w}^{+})$ is unstable and ${E}_{3}(0,\widetilde{y}, \widetilde{z}, \widetilde{u}, \widetilde{w})$ is stable. Note here that the values of ${u}^{+}$ and ${w}^{+}$ change depending on the values of ${R}_{\mathrm{U}}$ and $\chi$. See Supplementary Information, SI 3 for more details.

If the above conditions are satisfied, we observe evolutionary substitution (see Fig. 3), i.e., a mutant appears in the symbiont species, the resident phenotype of this species dies out, and the mutant coexists with the resident phenotype of the host by forming the consortium. If these sufficient conditions hold, this rules out the existence of the equilibrium point ${E}_{3}(0,\widetilde{y}, \widetilde{z}, \widetilde{u}, \widetilde{w})$. However, if these conditions are not satisfied, then ${E}_{3}$ may also exist and can also be locally asymptotically stable under some conditions. In other words, the mutant symbiont can coexist with the resident symbiont and the resident host (see Fig. 4). Conclusively, after the successful invasion, the possible outcomes of evolution are evolutionary substitution or coexistence of resident and mutant phenotypes (polymorphism). However, we limit our focus only to the case of evolutionary substitution because that is the scenario that gives fixation of consortia in the ecological model along with extinction of the resident phenotype of one of the species, which is our prime concern. The substitution of the resident phenotype by the mutant phenotype would eventually mean the emergence and fixation of the symbiotic merger in the ecological system.

**Figure 5:**
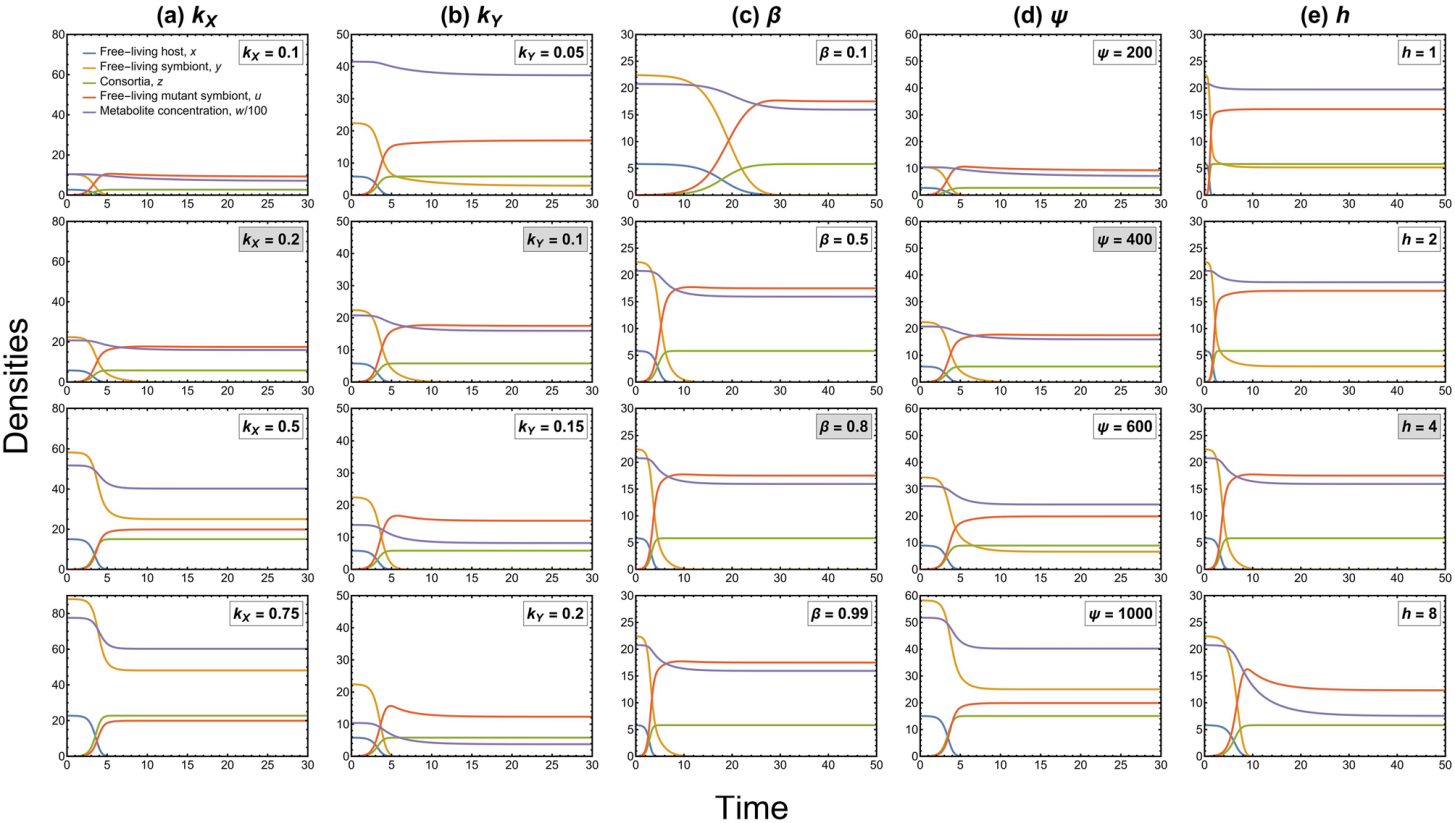
Time-series plots of the resident-mutant system (5D) showing the impact of several model parameters on the dynamics, keeping the rest of the parameters constant (values as in Table 1). The initial condition for all the plots is perturbed resident equilibrium ${E}_{1}\left({x}^{*},{y}^{*}, {0, 0, w}^{*}\right)$. The parameter values in the grey boxes correspond to the case as in the original dynamics used for numerical analysis in the Results Section. (**a**) A high consumption rate of the hosts ${(k}_{\mathrm{X}})$ facilitates the coexistence of symbiont phenotypes and hinders the possibility of evolutionary substitution. The higher the consumption, the higher the consortia density and concentration of its metabolic product. Consequently, the food available to the symbionts is high; thus, the two symbiont phenotypes can coexist. (**b**) A high consumption rate of symbionts ${(k}_{\mathrm{Y}})$ decreases the metabolite concentration and facilitates evolutionary substitution. The food available to the symbionts is low; thus, the two phenotypes of symbionts cannot coexist (competitive exclusion). (**c**) The rate of interaction of free-living mutant symbionts and free-living hosts $(\beta )$ does not affect the stability of the dynamics; however, stability is attained faster with higher interaction rates. (**d**) A high food concentration of hosts $(\psi )$ promotes the coexistence of symbiont phenotypes by increasing the metabolite concentration. (**e**) A high number of ectosymbionts on a host $(h)$ increases the possibility of evolutionary substitution and fixation of the consortia.

From a biological perspective, the analysis results of condition 1 suggest that the evolutionary substitution in our model happens when the ratio of cost of living to cost of reproduction of the mutant phenotype is less than that of the resident phenotype of the symbiont. Condition 2 could be considered as something that enables horizontal transmission in our model, i.e., the possibility of transfer of mutant symbionts from a consortium to a free-living host (when the rate of addition of free-living mutant symbionts into the habitat is high). Time-series plots corresponding to high and low values of parameter $\chi$ are shown in Figs. 3 and 4, respectively. Based on condition 2 for evolutionary substitution, we can also infer that high consumption rate of the hosts ${(k}_{\mathrm{X}})$ and high food concentration of hosts $(\psi )$ hinder the possibility of evolutionary substitution. In contrast, high consumption rate of symbionts ${(k}_{\mathrm{Y}})$ decreases the metabolite concentration and facilitates evolutionary substitution. This implies that the scarcity of the metabolic product in the habitat induces competitive exclusion between the two symbiont phenotypes. In addition, a high number of ectosymbionts on a host $(h)$ also facilitates the fixation of the consortia. Together with the result based on ${k}_{\mathrm{Y}}$, this suggests that small and highly metabolically active mutant symbionts are more likely to form consortia. We can also observe that the rate of interaction of free-living mutants and free-living hosts $(\beta )$ does not affect the stability of the dynamics; however, the stability in the dynamics is attained faster with high interaction rates. The impact of these model parameters has been shown with corresponding time-series plots in Fig. 5.

## Effectiveness of vertical transmission

If we suppose that the vertical transmission of the ectosymbionts on the consortia is not perfect, we have the following dynamics, where $\theta$ is the ‘effectiveness of vertical transmission’:

$$\dot{x}=x\left(\left(\frac{{{k}_{\mathrm{X}}\psi} -{C}_{\mathrm{X}}}{{R}_{\mathrm{X}}}\mathrm{ln}2-\frac{1}{{P}_{\mathrm{X}}}\right)-{a}_{1}(x+z)\right)-\frac{\beta xu}{h}+(1-\theta )\left(\frac{{{k}_{\mathrm{X}}\psi} -{C}_{\mathrm{X}}}{{R}_{\mathrm{X}}}\mathrm{ln}2-\frac{1}{{P}_{\mathrm{X}}}\right)z$$

$$\dot{y}=y\left(\frac{{{k}_{\mathrm{Y}}\frac{w}{V}}-{C}_{\mathrm{Y}}}{{R}_{\mathrm{Y}}}\mathrm{ln}2-\frac{1}{{P}_{\mathrm{Y}}}-{a}_{2}(y+u)\right)$$

$$\dot{z}=z\left(\theta \left(\frac{{{k}_{\mathrm{X}}\psi} -{C}_{\mathrm{X}}}{{R}_{\mathrm{X}}}\mathrm{ln}2-\frac{1}{{P}_{\mathrm{X}}}\right)-{a}_{1}\left(x+z\right)\right)+\frac{\beta xu}{h}$$

$$\dot{u}=u\left(\frac{{k}_{\mathrm{Y}}\frac{w}{V}-{C}_{\mathrm{U}}}{{R}_{\mathrm{U}}}\mathrm{ln}2-\frac{1}{{P}_{\mathrm{Y}}}-{a}_{2}(y+u)\right)-\beta xu+\chi z$$

$$\dot{w}={k}_{\mathrm{X}}\psi x{+{(1-k}_{\mathrm{Y}}h)k}_{\mathrm{X}}\psi z-\frac{{k}_{\mathrm{Y}}w}{V}\left(y+u\right)$$

**Figure 6:**
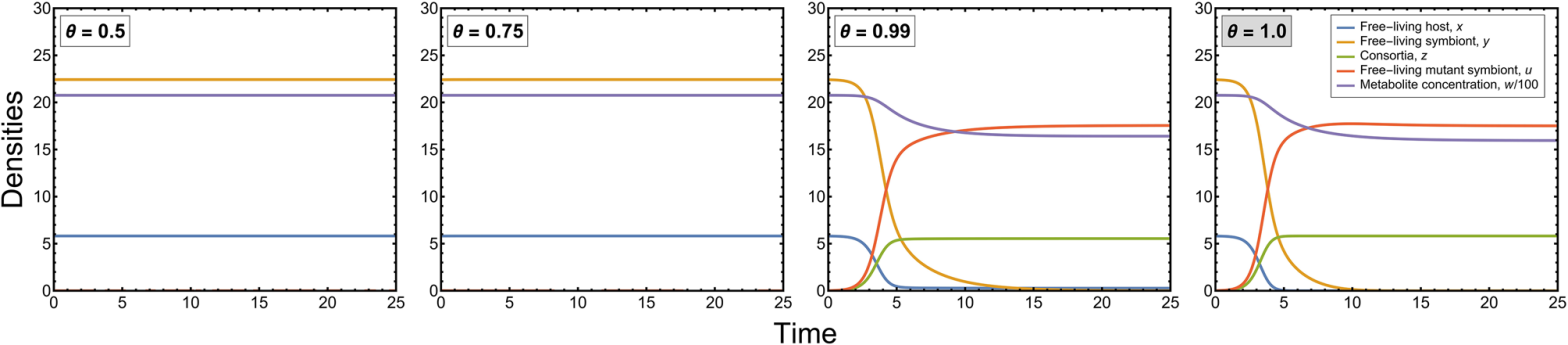
Time-series plots of the resident-mutant dynamics for different values of ‘effectiveness of vertical transmission, $\theta$’. The rest of the parameters are as in Fig. 3. The initial condition for all the plots is perturbed resident equilibrium ${E}_{1}\left({x}^{*},{y}^{*}, {0, 0, w}^{*}\right)$. The parameter value in the grey box corresponds to the case as in the original dynamics used for numerical analysis in the Results Section. The consortium density grows and stabilizes as the mutant symbiont replaces the resident symbiont when the vertical transmission is perfect or near perfect.

The parameter $\theta \in \left[\mathrm{0,1}\right]$ captures the probability that during the replication of consortia no symbiont gets to one of the daughter cells because of random assortment. $\theta =1$ corresponds to perfect vertical transmission, as assumed in Section "Coevolutionary system with ectosymbionts". The rest of the parameters and the variables of the above dynamics are as in Section "Coevolutionary system with ectosymbionts". Introducing the new parameter makes the model more general and open to more possibilities. We numerically analyzed the effect of this parameter on the dynamics. It was observed that the mutant symbiont and the consortia densities grow and stabilize when the vertical transmission is perfect or near perfect, i.e., $\theta \approx 1$ (see Fig. 6).

## Discussion, future directions, and biological examples

We found that if the ratio of cost of living to cost of reproduction of the mutant phenotype is less than that of the resident phenotype of the symbiont and the rate of addition of free-living mutant symbionts into the habitat is high (possibility of horizontal transmission), then the host-symbiont association or consortium is fixed by natural selection. We started with a commensalistic ecological interaction where the host’s metabolic product is food to the symbiont. We assume that a mutation occurs at this resident ecological equilibrium. The mutant symbionts can be joined by a novel structure to the external surface of the hosts, hence forming an ectocommensalistic association. Using a theoretical model, we analyzed the invasion of the mutant phenotype into the resident system. Even though both costs of living and reproduction of the mutant phenotype are greater than that of the resident phenotype, it is worthwhile to note that it is the ratio of the costs that matter, i.e., the additional costs of the connecting structure play a significant factor in the fixation of the consortia in the system.

Eukaryogenesis, as noted in the paper, is a complex process that requires many steps. The endosymbiosis of mitochondria, the origin of the nucleus, and the evolution of a sophisticated cytoskeleton, which ultimately allows for phagocytosis, eukaryotic genome organization, etc., are all steps in this transition. Each evolutionary step needs to be advantageous; thus, mostly, we need to assume small changes. We assumed one small change: the appearance (through mutation) of a molecule that can bind a commensal species to the host. This is one small step in the evolutionary path toward eukaryogenesis. As we are agnostic on the exact order of events, the host cell could have some eukaryote-like features, but none is needed for our model to work (for example, the host could have a nucleus, which would not change our results).

Furthermore, vertical transmission (when the symbionts are directly transferred from a host to its offspring) guarantees the association of the host and its symbiont in the next generation. One of the main consequences of vertical transmission is that the evolutionary success of the symbiont and its host is determined by that of the host-symbiont association^26^. Hence, the single mutation forms a new level of selection based on the fact that the symbionts can be vertically transmitted, and the consortia can be maintained, i.e., host and symbiont stick together and replicate. The main consequence of the stable physical contact between the host and symbiont species is that a new evolutionary unit and replicator type appears, namely the “consortium.” Studies show that the subsequent evolution of associations that probably started as a commensal one might lean towards parasitism in species with horizontal transmission, while vertical transmission promotes mutualism^27, 28^. Evolution from horizontal transmission and parasitism towards vertical transmission and mutualism has also been discussed previously^29, 30^. Thus, evidence supports the argument that the mode of transmission highly influences evolution toward mutualism or parasitism. In other words, vertical transmission opens the door to the coevolution of the host-symbiont pair in the direction of mutualism. One of the essential steps in the evolution of intimate and obligate symbiosis between primitive unicellular organisms could be a mutation that allows vertical transmission, just as we considered. Thus, the emergence of vertical transmission should be one of the critical questions in the evolution of symbiosis.

Conclusively, after the fixation of the host-symbiont association in the system, the main direction for evolutionary mechanisms is that the association can evolve to be mutualistic. For example, at some point in time, excess concentrations of the metabolic waste product of the host in its proximity turn out to be toxic to itself, and the symbiont plays a significant role in reducing this concentration below a critical level, thereby helping the host in its survival. In this scenario, mutualism can emerge because each member benefits the other. This can be subject to further study with a toxicity-dependent bounding of species densities. Another possibility is the evolution of cyclic syntrophy, i.e., as a result of a mutation on the host, it can use at least one of the metabolic products of its ectosymbionts (considering the symbiont has not changed itself by other mutations). This is similar to the concept of evolutionary replacement discussed by Cressman and Garay^24^. Unlike evolutionary substitution, evolutionary replacement requires that there are mutant phenotypes in each of the species. In this scenario too, a costless mutualism can emerge. Moreover, since the host and the symbiont are different species, we can assume that the metabolic abilities of the species are quite different, and thereby the symbiont can potentially produce metabolic products that the host is incapable of producing itself and can use as food. In the case of mutualistic interactions, vertical transmission is more evolutionarily rational^27^ since multi-level selection can enforce better association. In other words, such associations have a beneficial connection for both the host and its vertically transmitted symbiont; thus, the reproduction rate of this association should be higher than other possibilities. Hence, the mutations which force vertical transmission should invade the population.

We assumed that the host was totally impassive toward the symbionts to make the model more straightforward. Also, we made some assumptions on the parameters of the model to maintain tractability. However, relaxing some of the assumptions in the model could be an immediate extension of the work. For instance, a responsive host that detects the presence of ectosymbionts, whose feeding is impacted by the ectosymbionts. Our model can be extended further with appropriate modifications to analyze endosymbiotic associations as well. Moreover, vertical transmission is more likely for the endosymbiont than for the ectosymbiont. For instance, suppose the ectosymbiont can move into the host cell by engulfment via phagocytosis or slowly increasing surface contact during ectosymbiotic syntrophy or by bacterial invasion^13^. After this, the “consensus” of the metabolisms of the host and its internalized symbionts can lead to the emergence of endosymbiosis, for instance, in the case of the mitochondrial ancestor^8, 9, 12^.

Our study was motivated by the origin of eukaryotes, a major evolutionary transition, and the emergence of a new level of individuality. We try to provide a theoretical analysis of a hypothesis on the symbiogenetic origins of multi-species intimate associations, particularly a eukaryotic cell. The concept of syntrophy is prevalent among the several hypotheses trying to deepen the understanding of complex evolutionary transitions^13^, hence the need to model it mathematically. The novel growth rate introduced in the model can be utilized to structure interactions based on syntrophy and develop metabolite-dependent ecological dynamics. Utilizing the concept of mutant invasion to form an ectocommensalism in a dynamical setup to understand symbiosis is also a novel approach. The paper attempts to model (as a dynamical system) syntrophic interactions leading to the formation of symbiotic behavior between primitive unicellular organisms.

Ectosymbiosis is indeed common in nature, even among prokaryotes. Recently, several ectosymbionts belonging to the Candidate Phyla Radiation clade of Bacteria and DPANN clade of Archaea have been identified in ectosymbiosis with a Bacterial or Archaeal host^31–33^. It demonstrates that prokaryotes are capable of being both the host and the symbiont in these associations. *Nanoarchaeum equitans*^34^ is the most well-studied symbiont (or parasite; the exact nature of the association is still debated), which is attached to cells of *Ignicoccus hospitalis* as its host. Since their discovery, other nanoarchaeal ectosymbionts were identified, such as *Nanoclepta minutus* and its host *Zestosphaera tikiterensis*^35^, *Nanopusillus acidilobi* and its host an *Acidilobus* species^36^, and *Nanobdella aerobiophila* and its host, *Metallosphaera sedula*^37^. Similar symbiont-host pairs were found among the other small-celled Archaeal phylum, the Nanohaloarchaeota: *Nanohalobium constans* with a *Halomicrobium* species^38^, *Micrarchaeum harzensis* with *Scheffleriplasma hospitalis*^39^, and *Nanohaloarchaeum antarcticus* with its host *Halorubrum lacusprofundi*^40^. From the Candidate Phyla Radiation, *Saccharibacteria* (TM7) should be mentioned as they are ectosymbionts of various host bacteria, including *Actinomyces* species^41, 42^ living in the human oral microbiome. The interaction is probably initiated by the symbiont, as it is the symbiont that cannot live without its host. The host can live (can be cultured) without its ectosymbiont. Ultrastructural analysis also uncovers cell surface structures attaching to and penetrating the outer layers of the host^38, 39, 43^, and in many cases, a pili-like structure is observed at the attachment site^44–46^. Pili is an established way for archaea^45^ and bacteria^44^ to attach to another cell. Still, the exact way the ectosymbiont attaches to its host is unknown. Studies have implicated various proteins, such as the WD-40 protein family of *Ignicoccus hospitalis*^46^, SPEARE proteins of *Nanohaloarchaea*^40^, or proteins involved in type 4 pili formation^47^ in the case of Nanoarchaeota. Proteomic analysis of the contact site of the two Archaea revealed some putative transporters and membrane proteins^48^, which other studies, mostly genetic, corroborate^49^. The attachment to the host is quite strong, and a given host cell is covered by clones derived from a single symbiotic cell^47^; thus, the ectosymbionts proliferate on the host cell’s surface. In the end, there could be several symbionts on any host cell: around 10 *Nanoarchaeum equitans* on *Ignicoccus hospitalis*^50^, between 1 and 10 *Nanopusillus acidilobi* on *Acidilobus* species^36^, and about 4–5 (up to 17) *Nanohalobium constans* on *Halomicrobium* species. Our model is built on these observations. We hope our attempt opens a different perspective of modeling and analyzing unicellular symbiosis.

### Supplementary Information

Supplementary Information 1.

Supplementary Information 2.

## Data Availability

All data generated or analyzed during this study are included in the article (and its Supplementary Information files).

## Code availability

The codes to reproduce all the data and figures generated or analyzed during the study are provided as a separate Supplementary file (see SI 5).
