# Supplementary material for: Genesis of ectosymbiotic features based on commensalistic syntrophy
Source: Sci Rep. 2024 Jan 16;14:1366. doi: 10.1038/s41598-023-47211-8 (PMC10791676; doi:10.1038/s41598-023-47211-8)
Supplement: Supplementary file 1 — Supplementary Information 1. [file 41598_2023_47211_MOESM1_ESM.pdf]

Genesis of ectosymbiotic features based on commensalistic syntrophy

Equilibrium points

5-D Resident-Mutant system

```
ClearAll["Global`*"];

f1[x_, y_, z_, u_, w_] := x*(\[Alpha] - a1*x - a1*z - (\[Beta]/h)*u);
f2[x_, y_, z_, u_, w_] := y*(\[Eta]*w - \[Rho] - a2*y - a2*u);
f3[x_, y_, z_, u_, w_] := z*(\[Alpha] - a1*x - a1*z) + (\[Beta]/h)*x*u;
f4[x_, y_, z_, u_, w_] :=  u*(\[Mu]*w - \[Sigma] - a2*y - a2*u - \[Beta]*x) + \[Chi]*z;
f5[x_, y_, z_, u_, w_] := \[Phi]*x + \[Kappa]*\[Phi]*z - \[Xi]*w*y - \[Xi]*w*u;
 sol =  Simplify[Solve[{f1[x, y, z, u, w] == 0, f2[x, y, z, u, w] == 0,  f3[x, y, z, u, w] == 0,
 f4[x, y, z, u, w] == 0, f5[x, y, z, u, w] == 0}, {x, y, z, u, w}]]
```

3-D Resident-only system

```
ClearAll["Global`*"];

f1[x_, y_, w_] := x*(\[Alpha] - a1*x);
f2[x_, y_, w_] := y*(\[Eta]*w - \[Rho] - a2*y);
f3[x_, y_, w_] := \[Phi]*x - \[Xi]*w*y;
sol1 = Simplify[ Solve[{f1[x, y, w] == 0, f2[x, y, w] == 0, f3[x, y, w] == 0}, {x, y, w}]]
```

3-D Mutant-only system

```
ClearAll["Global`*"];
```

```
f1[z_, u_, w_] := z*(\[Alpha] - a1*z);
f2[z_, u_, w_] := u*(\[Mu]*w - \[Sigma] - a2*u) + \[Chi]*z;
f3[z_, u_, w_] := \[Kappa]*\[Phi]*z - \[Xi]*w*u;
sol2 = Simplify[ Solve[{f1[z, u, w] == 0, f2[z, u, w] == 0, f3[z, u, w] == 0}, {z, u, w}]]
```

Phase portraits (Figure 2)

```
ClearAll["Global`*"];

p1 = Show[ StreamPlot3D[{x *(17.46 - 3*x), y *(2.3*w - 3.106 - 2*y),
    80*x - 1*w*y}, {x, 0, 100}, {y, 0, 100}, {w, 0, 100},
   StreamColorFunction -> None, StreamStyle -> Darker[Blue],
   StreamMarkers -> "Arrow", StreamPoints -> Coarse,
   AxesStyle -> Directive[Black, 12], ImageSize -> {340, 360},
   PlotLabel -> Style["(a)", FontFamily -> "Arial", Bold, Black, 20],
   ImagePadding -> {{30, 10}, {30, 30}}],
  Graphics3D[{Red, PointSize[0.02], Point[{5.82, 22.43, 20.76}]}]];

labelx1 = Style[Rotate["Free-living host, \!\(\*
StyleBox[\"x\",\nFontSlant->\"Italic\"]\)", -22 Degree],
  FontFamily -> "Arial",  14, Black];
labely1 = Style[Rotate["Free-living symbiont, \!\(\*
StyleBox[\"y\",\nFontSlant->\"Italic\"]\)", 35 Degree],
  FontFamily -> "Arial",  14, Black];
labelz1 = Style[Rotate["Metabolite conc., \!\(\*
StyleBox[\"w\",\nFontSlant->\"Italic\"]\)/100 ", 90 Degree],
  FontFamily -> "Arial", 14, Black];

pt1 = Scaled[{0.37, 0.1}];
pt2 = Scaled[{0.27, 0.78}];
```

```
pt3 = Scaled[{0.07, 0.4}];

fig2a = Overlay[{p1,
    Graphics[{}, AspectRatio -> (360)/(340),
     ImageSize -> {340, 360}, ImagePadding -> 0,
     Epilog -> {Dynamic[Locator[Dynamic[pt1], labelx1]],
     Dynamic[Locator[Dynamic[pt2], labely1]],
     Dynamic[Locator[Dynamic[pt3], labelz1]]}]}, All, 2];

FIGURE2a = fig2a /. Locator[x_, y_] :> Inset[y, x] /. Dynamic :> Identity;

p2 = Show[
  StreamPlot3D[{z*(17.46 - 3*z), u*(1.7*w - 2.586 - 2*u) + 30*z,
    48*z - 1*w*u}, {z, 0, 100}, {u, 0, 100}, {w, 0, 100},
   StreamColorFunction -> None, StreamStyle -> Darker[Blue],
   StreamMarkers -> "Arrow", StreamPoints -> Coarse,
   AxesStyle -> Directive[Black, 12], ImageSize -> {340, 360},
   PlotLabel -> Style["(b)", FontFamily -> "Arial", Bold, Black, 20],
   ImagePadding -> {{30, 10}, {30, 30}}],
  Graphics3D[{Red, PointSize[0.02], Point[{5.82, 17.51, 15.95}]}]];

labelx2 = Style[Rotate["Consortium, \!\(\*
StyleBox[\"z\",\nFontSlant->\"Italic\"]\)", -22 Degree],
  FontFamily -> "Arial",  14, Black];
labely2 = Style[Rotate["Mutant symbiont, \!\(\*
StyleBox[\"u\",\nFontSlant->\"Italic\"]\)", 35 Degree],
  FontFamily -> "Arial",  14, Black];
labelw2 = Style[Rotate["Metabolite conc., \!\(\*
StyleBox[\"w\",\nFontSlant->\"Italic\"]\)/100 ", 90 Degree],
  FontFamily -> "Arial", 14, Black];

pt4 = Scaled[{0.37, 0.1}];
```

```
pt5 = Scaled[{0.27, 0.78}];
pt6 = Scaled[{0.07, 0.4}];

fig2b = Overlay[{p2,
    Graphics[{}, AspectRatio -> (360)/(340),
     ImageSize -> {340, 360}, ImagePadding -> 0,
     Epilog -> {Dynamic[Locator[Dynamic[pt4], labelx2]],
       Dynamic[Locator[Dynamic[pt5], labely2]],
       Dynamic[Locator[Dynamic[pt6], labelw2]]}]}, All, 2];

FIGURE2b = fig2b /. Locator[x_, y_] :> Inset[y, x] /. Dynamic :> Identity;

FIGURE2 = Grid[{{FIGURE2a, FIGURE2b}}]
```

Evolutionary substitution (Figure 3)

```
ClearAll["Global`*"];

a1 = 3; a2 = 2; \[Psi] = 400; kx = 0.2; Cx = 4; Rx = 3; Px = 10; ky = 0.1; Ry = 0.3; V = 10; Cy = 1.2; Py = 3; \[Beta] = 0.8; h = 4; \[Chi] = 30; Ru = 0.4; Cu = 1.3;

\[Alpha] = (((kx*\[Psi] - Cx)*(N[Log[2]]))/Rx) - (1/Px);
\[Eta] = (ky*(N[Log[2]]))/(V*Ry);
\[Rho] = ((Cy*(N[Log[2]]))/Ry) + (1/Py);
\[Phi] = kx*\[Psi];
\[Xi] = ky/V;
\[Kappa] = 1 - ky*h;
\[Mu] = (ky*(N[Log[2]]))/(V*Ru);
\[Sigma] = ((Cu*(N[Log[2]]))/Ru) + (1/Py);

q1 = Plot[Evaluate[NDSolveValue[{
    x'[t] == x[t]*(\[Alpha] - a1*x[t] - a1*z[t] - (\[Beta]/h)*u[t]),
```

```
    y'[t] == y[t]*(\[Eta]*w[t] - \[Rho] - a2*y[t] - a2*u[t]),
    z'[t] == z[t]*(\[Alpha] - a1*x[t] - a1*z[t]) + (\[Beta]/h)*x[t]*u[t],
    u'[t] == u[t]*(\[Mu]*w[t] - \[Sigma] - a2*y[t] - a2*u[t] - \[Beta]*x[t]) + \[Chi]*z[t],
    w'[t] == \[Phi]*x[t] + \[Kappa]*\[Phi]*z[t] - \[Xi]*w[t]*y[t] - \[Xi]* w[t]*u[t],
    x[0] == 0.01, y[0] == 0.01, z[0] == 0.01, u[0] == 0.01,
    w[0] == 0.01}, {x[t], y[t], z[t], u[t], (w[t]/100)}, {t, 0, 100}]], {t,
   0, 100}, PlotStyle -> Directive[Thickness[0.008], 12],
  PlotRange -> {{0, 30}, {0, 30}},
  PlotLabel -> Style["(a)", FontFamily -> "Arial", Bold, Black, 20],
  PlotLegends -> Placed[LineLegend[Automatic, {Style["Free-living host, \!\(\*
StyleBox[\"x\",\nFontSlant->\"Italic\"]\)", FontFamily -> "Arial", 10, Black],
     Style["Free-living symbiont, \!\(\*
StyleBox[\"y\",\nFontSlant->\"Italic\"]\)", FontFamily -> "Arial", 10, Black],
     Style["Consortia, \!\(\*
StyleBox[\"z\",\nFontSlant->\"Italic\"]\)", FontFamily -> "Arial", 10, Black],
     Style["Free-living mutant symbiont, \!\(\*
StyleBox[\"u\",\nFontSlant->\"Italic\"]\)", FontFamily -> "Arial", 10, Black],
     Style["Metabolite concentration, \!\(\*
StyleBox[\"w\",\nFontSlant->\"Italic\"]\)/100", FontFamily -> "Arial", 10,
     Black]}, LegendLayout -> (Grid[#, Spacings -> {.5, .1},
       Alignment -> Left] &),
    LegendFunction -> (Framed[#, Background -> White, FrameMargins -> 1.0,
       FrameStyle -> AbsoluteThickness[0.5],
       RoundingRadius -> 0] &)], {{0.68, 0.98}, {0.5, 1.0}}],
  Frame -> True,
  FrameLabel -> {Style["Time", FontFamily -> "Arial", Black, 18],
   Style["Densities", FontFamily -> "Arial", Black, 18]},
  FrameStyle -> Directive[Black, Thickness[0.006], 14], RotateLabel -> True,
  RotateLabel -> True, ImageSize -> {400, 300}, AspectRatio -> 0.75];

q2 = Plot[Evaluate[NDSolveValue[{
    x'[t] == x[t]*(\[Alpha] - a1*x[t] - a1*z[t] - (\[Beta]/h)*u[t]),
```

```
    y'[t] == y[t]*(\[Eta]*w[t] - \[Rho] - a2*y[t] - a2*u[t]),
    z'[t] == z[t]*(\[Alpha] - a1*x[t] - a1*z[t]) + (\[Beta]/h)*x[t]*u[t],
    u'[t] ==  u[t]*(\[Mu]*w[t] - \[Sigma] - a2*y[t] -  a2*u[t] - \[Beta]*x[t]) + \[Chi]*z[t],
    w'[t] == \[Phi]*x[t] + \[Kappa]*\[Phi]*z[t] - \[Xi]*w[t]*y[t] - \[Xi]* w[t]*u[t],
    x[0] == 5.82, y[0] == 22.43, z[0] == 0.01, u[0] == 0.01,
    w[0] == 2076}, {x[t], y[t], z[t], u[t], (w[t]/100)}, {t, 0, 100}]], {t,
   0, 100}, PlotStyle -> Directive[Thickness[0.008], 12],
  PlotRange -> {{0, 30}, {0, 30}},
  PlotLabel -> Style["(b)", FontFamily -> "Arial", Bold, Black, 20],
  Frame -> True,
  FrameLabel -> {Style["Time", FontFamily -> "Arial", 18, Black],
    Style["Densities", FontFamily -> "Arial", 18, Black]},
  FrameStyle -> Directive[Black, Thickness[0.006], 14], RotateLabel -> True,
  RotateLabel -> True, ImageSize -> {400, 300}, AspectRatio -> 0.75];

FIG3 = Grid[{{q1, q2}}];

FIGURE3 =
 Labeled[FIG3, {Style["Evolutionary substitution", FontFamily -> "Arial",
    Bold, 20, Black]}, {Top}, Spacings -> {0.1, 1.0}]
```

Polymorphism (Figure 4)

```
ClearAll["Global`*"];

a1 = 3; a2 = 2; \[Psi] = 400; kx = 0.2; Cx = 4; Rx = 3; Px = 10; ky = 0.1; Ry = 0.3; V = 10; Cy =
1.2; Py = 3; \[Beta] = 0.8; h = 4; \[Chi] = 5; Ru = 0.32; Cu = 1.3;

\[Alpha] = (((kx*\[Psi] - Cx)*(N[Log[2]]))/Rx) - (1/Px);
\[Eta] = (ky*(N[Log[2]]))/(V*Ry);
\[Rho] = ((Cy*(N[Log[2]]))/Ry) + (1/Py);
\[Phi] = kx*\[Psi];
```

```
\[Xi] = ky/V;
\[Kappa] = 1 - ky*h;
\[Mu] = (ky*(N[Log[2]]))/(V*Ru);
\[Sigma] = ((Cu*(N[Log[2]]))/Ru) + (1/Py);

q2 = Plot[Evaluate[NDSolveValue[{
    x'[t] == x[t]*(\[Alpha] - a1*x[t] - a1*z[t] - (\[Beta]/h)*u[t]),
    y'[t] == y[t]*(\[Eta]*w[t] - \[Rho] - a2*y[t] - a2*u[t]),
    z'[t] == z[t]*(\[Alpha] - a1*x[t] - a1*z[t]) + (\[Beta]/h)*x[t]*u[t],
    u'[t] ==  u[t]*(\[Mu]*w[t] - \[Sigma] - a2*y[t] -  a2*u[t] - \[Beta]*x[t]) + \[Chi]*z[t],
    w'[t] == \[Phi]*x[t] + \[Kappa]*\[Phi]*z[t] - \[Xi]*w[t]*y[t] - \[Xi]*  w[t]*u[t],
    x[0] == 5.82, y[0] == 22.43, z[0] == 0.01, u[0] == 0.01,
    w[0] == 2076}, {x[t], y[t], z[t], u[t], (w[t]/100)}, {t, 0, 100}]], {t,
   0, 100}, PlotStyle -> Directive[Thickness[0.008], 12],
  PlotRange -> {{0, 50}, {0, 30}},
  PlotLabel -> Style["(a)", FontFamily -> "Arial", Bold, Black, 20],
  PlotLegends -> Placed[LineLegend[Automatic, {Style["Free-living host, \!\(\*
StyleBox[\"x\",\nFontSlant->\"Italic\"]\)", FontFamily -> "Arial", 10, Black],
      Style["Free-living symbiont, \!\(\*
StyleBox[\"y\",\nFontSlant->\"Italic\"]\)", FontFamily -> "Arial", 10, Black],
      Style["Consortia, \!\(\*
StyleBox[\"z\",\nFontSlant->\"Italic\"]\)", FontFamily -> "Arial", 10, Black],
      Style["Free-living mutant symbiont, \!\(\*
StyleBox[\"u\",\nFontSlant->\"Italic\"]\)", FontFamily -> "Arial", 10, Black],
      Style["Metabolite concentration, \!\(\*
StyleBox[\"w\",\nFontSlant->\"Italic\"]\)/100", FontFamily -> "Arial", 10,
      Black]}, LegendLayout -> (Grid[#, Spacings -> {.5, .1},
        Alignment -> Left] &),
    LegendFunction -> (Framed[#, Background -> White, FrameMargins -> 1.0,
        FrameStyle -> AbsoluteThickness[0.5],
        RoundingRadius -> 0] &)], {{0.68, 0.98}, {0.5, 1.0}}],
  Frame -> True,
```

```
  FrameLabel -> {Style["Time", FontFamily -> "Arial", 18, Black],
    Style["Densities", FontFamily -> "Arial", 18, Black]},
  FrameStyle -> Directive[Black, Thickness[0.006], 14], RotateLabel -> True,
  RotateLabel -> True, ImageSize -> {400, 300}, AspectRatio -> 0.75];

q3 = Plot[Evaluate[NDSolveValue[{
     x'[t] == x[t]*(\[Alpha] - a1*x[t] - a1*z[t] - (\[Beta]/h)*u[t]),
     y'[t] == y[t]*(\[Eta]*w[t] - \[Rho] - a2*y[t] - a2*u[t]),
     z'[t] == z[t]*(\[Alpha] - a1*x[t] - a1*z[t]) + (\[Beta]/h)*x[t]*u[t],
     u'[t] ==  u[t]*(\[Mu]*w[t] - \[Sigma] - a2*y[t] -  a2*u[t] - \[Beta]*x[t]) + \[Chi]*z[t],
     w'[t] == \[Phi]*x[t] + \[Kappa]*\[Phi]*z[t] - \[Xi]*w[t]*y[t] - \[Xi]* w[t]*u[t],
     x[0] == 0.01, y[0] == 0.01, z[0] == 5.82, u[0] == 17.04,
     w[0] == 1640}, {x[t], y[t], z[t], u[t], (w[t]/100)}, {t, 0, 100}]], {t,
   0, 100}, PlotStyle -> Directive[Thickness[0.008], 12],
  PlotRange -> {{0, 50}, {0, 30}},
  PlotLabel -> Style["(b)", FontFamily -> "Arial", Bold, Black, 20],
  Frame -> True,
  FrameLabel -> {Style["Time", FontFamily -> "Arial", 18, Black],
    Style["Densities", FontFamily -> "Arial", 18, Black]},
  FrameStyle -> Directive[Black, Thickness[0.006], 14], RotateLabel -> True,
  RotateLabel -> True, ImageSize -> {400, 300}, AspectRatio -> 0.75];

FIG4 = Grid[{{q2, q3}}];

FIGURE4 =
 Labeled[FIG4, {Style["Polymorphism", FontFamily -> "Arial", Bold, 20,
    Black]}, {Top}, Spacings -> {0.1, 1.0}]
```

Impact of host consumption rate, kx (Figure 5a)

```
ClearAll["Global`*"];
```

```
a1 = 3; a2 = 2; \[Psi] = 400; V = 10; Cx = 4; Rx = 3; Px = 10; ky = 0.1; Ry = 0.3; Cy = 1.2; Py =
3; \[Beta] = 0.8; h = 4; \[Chi] = 30; Ru = 0.4; Cu = 1.3;

kx  = 0.1

\[Alpha] = (((0.1*\[Psi] - Cx)*(N[Log[2]]))/Rx) - (1/Px);
\[Eta] = (ky*(N[Log[2]]))/(V*Ry);
\[Rho] = ((Cy*(N[Log[2]]))/Ry) + (1/Py);
\[Phi] = 0.1*\[Psi];
\[Xi] = ky/V;
\[Kappa] = 1 - ky*h;
\[Mu] = (ky*(N[Log[2]]))/(V*Ru);
\[Sigma] = ((Cu*(N[Log[2]]))/Ru) + (1/Py);

r1 = Plot[Evaluate[NDSolveValue[{
    x'[t] == x[t]*(\[Alpha] - a1*x[t] - a1*z[t] - (\[Beta]/h)*u[t]),
    y'[t] == y[t]*(\[Eta]*w[t] - \[Rho] - a2*y[t] - a2*u[t]),
    z'[t] == z[t]*(\[Alpha] - a1*x[t] - a1*z[t]) + (\[Beta]/h)*x[t]*u[t],
    u'[t] ==  u[t]*(\[Mu]*w[t] - \[Sigma] - a2*y[t] -  a2*u[t] - \[Beta]*x[t]) + \[Chi]*z[t],
    w'[t] == \[Phi]*x[t] + \[Kappa]*\[Phi]*z[t] - \[Xi]*w[t]*y[t] - \[Xi]* w[t]*u[t],
    x[0] == 2.74, y[0] == 10.50, z[0] == 0.01, u[0] == 0.01,
    w[0] == 1043}, {x[t], y[t], z[t], u[t], (w[t]/100)}, {t, 0, 100}]], {t,
   0, 100}, PlotStyle -> Directive[Thickness[0.008], 12],
  PlotRange -> {{0, 30}, {0, 80}},
  PlotLegends -> Placed[LineLegend[Automatic, {Style["Free-living host, \!\(\*
StyleBox[\"x\",\nFontSlant->\"Italic\"]\)", FontFamily -> "Arial", 18, Black],
     Style["Free-living symbiont, \!\(\*
StyleBox[\"y\",\nFontSlant->\"Italic\"]\)", FontFamily -> "Arial", 18, Black],
     Style["Consortia, \!\(\*
StyleBox[\"z\",\nFontSlant->\"Italic\"]\)", FontFamily -> "Arial", 18, Black],
     Style["Free-living mutant symbiont, \!\(\*
```

```
StyleBox[\"u\",\nFontSlant->\"Italic\"]\)", FontFamily -> "Arial", 18, Black],
        Style["Metabolite concentration, \!\(\*
StyleBox[\"w\",\nFontSlant->\"Italic\"]\)/100", FontFamily -> "Arial", 18,
        Black]}, LegendLayout -> (Grid[#, Spacings -> {.5, .5},
          Alignment -> Left] &)], {{0.45, 0.95}, {0.5, 1.0}}], Frame -> True,
   FrameStyle -> Directive[Black, Thickness[0.006], 18],
   ImageSize -> {400, 300}, AspectRatio -> 0.75];

kx  = 0.2

\[Alpha] = (((0.2*\[Psi] - Cx)*(N[Log[2]]))/Rx) - (1/Px);
\[Eta] = (ky*(N[Log[2]]))/(V*Ry);
\[Rho] = ((Cy*(N[Log[2]]))/Ry) + (1/Py);
\[Phi] = 0.2*\[Psi];
\[Xi] = ky/V;
\[Kappa] = 1 - ky*h;
\[Mu] = (ky*(N[Log[2]]))/(V*Ru);
\[Sigma] = ((Cu*(N[Log[2]]))/Ru) + (1/Py);

r2 = Plot[Evaluate[NDSolveValue[{
      x'[t] == x[t]*(\[Alpha] - a1*x[t] - a1*z[t] - (\[Beta]/h)*u[t]),
      y'[t] == y[t]*(\[Eta]*w[t] - \[Rho] - a2*y[t] - a2*u[t]),
      z'[t] == z[t]*(\[Alpha] - a1*x[t] - a1*z[t]) + (\[Beta]/h)*x[t]*u[t],
      u'[t] ==  u[t]*(\[Mu]*w[t] - \[Sigma] - a2*y[t] -  a2*u[t] - \[Beta]*x[t]) + \[Chi]*z[t],
      w'[t] == \[Phi]*x[t] + \[Kappa]*\[Phi]*z[t] - \[Xi]*w[t]*y[t] - \[Xi]*w[t]*u[t],
      x[0] == 5.82, y[0] == 22.43, z[0] == 0.01, u[0] == 0.01,
      w[0] == 2076}, {x[t], y[t], z[t], u[t], (w[t]/100)}, {t, 0, 100}]], {t,
    0, 100}, PlotStyle -> Directive[Thickness[0.008], 12],
   PlotRange -> {{0, 30}, {0, 80}}, Frame -> True,
   FrameStyle -> Directive[Black, Thickness[0.006], 18],
   ImageSize -> {400, 300}, AspectRatio -> 0.75];
```

kx = 0.5

```
\[Alpha] = (((0.5*\[Psi] - Cx)*(N[Log[2]]))/Rx) - (1/Px);
\[Eta] = (ky*(N[Log[2]]))/(V*Ry);
\[Rho] = ((Cy*(N[Log[2]]))/Ry) + (1/Py);
\[Phi] = 0.5*\[Psi];
\[Xi] = ky/V;
\[Kappa] = 1 - ky*h;
\[Mu] = (ky*(N[Log[2]]))/(V*Ru);
\[Sigma] = ((Cu*(N[Log[2]]))/Ru) + (1/Py);

r3 = Plot[Evaluate[NDSolveValue[{
    x'[t] == x[t]*(\[Alpha] - a1*x[t] - a1*z[t] - (\[Beta]/h)*u[t]),
    y'[t] == y[t]*(\[Eta]*w[t] - \[Rho] - a2*y[t] - a2*u[t]),
    z'[t] == z[t]*(\[Alpha] - a1*x[t] - a1*z[t]) + (\[Beta]/h)*x[t]*u[t],
    u'[t] == u[t]*(\[Mu]*w[t] - \[Sigma] - a2*y[t] -  a2*u[t] - \[Beta]*x[t]) + \[Chi]*z[t],
    w'[t] == \[Phi]*x[t] + \[Kappa]*\[Phi]*z[t] - \[Xi]*w[t]*y[t] - \[Xi]* w[t]*u[t],
    x[0] == 15.06, y[0] == 58.22, z[0] == 0.01, u[0] == 0.01,
    w[0] == 5174}, {x[t], y[t], z[t], u[t], (w[t]/100)}, {t, 0, 100}]], {t,
   0, 100}, PlotStyle -> Directive[Thickness[0.008], 12],
  PlotRange -> {{0, 30}, {0, 80}}, Frame -> True,
  FrameStyle -> Directive[Black, Thickness[0.006], 18],
  ImageSize -> {400, 300}, AspectRatio -> 0.75];
```

kx = 0.75

```
\[Alpha] = (((0.75*\[Psi] - Cx)*(N[Log[2]]))/Rx) - (1/Px);
\[Eta] = (ky*(N[Log[2]]))/(V*Ry);
\[Rho] = ((Cy*(N[Log[2]]))/Ry) + (1/Py);
\[Phi] = 0.75*\[Psi];
\[Xi] = ky/V;
\[Kappa] = 1 - ky*h;
```

```
\[Mu] = (ky*(N[Log[2]]))/(V*Ru);
\[Sigma] = ((Cu*(N[Log[2]]))/Ru) + (1/Py);

r4 = Plot[Evaluate[NDSolveValue[{
    x'[t] == x[t]*(\[Alpha] - a1*x[t] - a1*z[t] - (\[Beta]/h)*u[t]),
    y'[t] == y[t]*(\[Eta]*w[t] - \[Rho] - a2*y[t] - a2*u[t]),
    z'[t] == z[t]*(\[Alpha] - a1*x[t] - a1*z[t]) + (\[Beta]/h)*x[t]*u[t],
    u'[t] == u[t]*(\[Mu]*w[t] - \[Sigma] - a2*y[t] - a2*u[t] - \[Beta]*x[t]) + \[Chi]*z[t],
    w'[t] == \[Phi]*x[t] + \[Kappa]*\[Phi]*z[t] - \[Xi]*w[t]*y[t] - \[Xi]*w[t]*u[t],
    x[0] == 22.76, y[0] == 88.05, z[0] == 0.01, u[0] == 0.01,
    w[0] == 7756}, {x[t], y[t], z[t], u[t], (w[t]/100)}, {t, 0, 100}]], {t,
  0, 100}, PlotStyle -> Directive[Thickness[0.008], 12],
 PlotRange -> {{0, 30}, {0, 90}}, Frame -> True,
 FrameStyle -> Directive[Black, Thickness[0.006], 18],
 ImageSize -> {400, 300}, AspectRatio -> 0.75];
```

Combined

```
Figurekx =
 ResourceFunction["PlotGrid"][{{r1}, {r2}, {r3}, {r4}}, Spacings -> 40,
  PlotLabel ->
   Style["(a) \!\(\*SubscriptBox[\(k\), \(X\)]\)", FontFamily -> "Arial", 36,
    Bold, Black],
  FrameStyle -> Directive[Black, FontFamily -> "Arial", FontSize -> 40],
  PlotLabels -> { Placed[Framed[Style["\!\(\*SubscriptBox[\(k\), \(X\)]\) = 0.1",
      FontFamily -> "Arial", 24, Bold, Black]], {Right, Top}],
    Placed[Framed[ Style["\!\(\*SubscriptBox[\(k\), \(X\)]\) = 0.2",
      FontFamily -> "Arial", 24, Bold, Black],
     Background -> LightGray], {Right, Top}],
    Placed[Framed[Style["\!\(\*SubscriptBox[\(k\), \(X\)]\) = 0.5",
      FontFamily -> "Arial", 24, Bold, Black]], {Right, Top}],
    Placed[Framed[Style["\!\(\*SubscriptBox[\(k\), \(X\)]\) = 0.75",
```

```
    FontFamily -> "Arial", 24, Bold, Black]], {Right, Top}] }
  , ImageSize -> {400, 1200}]
```

Impact of symbiont consumption rate, ky (Figure 5b)

```
ClearAll["Global`*"];

a1 = 3; a2 = 2; \[Psi] = 400; V = 10; Cx = 4; Rx = 3; Px = 10; kx = 0.2; Ry = 0.3; Cy = 1.2; Py =
3; \[Beta] = 0.8; h = 4; \[Chi] = 30; Ru = 0.4; Cu = 1.3;
```

ky = 0.05

```
\[Alpha] = (((kx*\[Psi] - Cx)*(N[Log[2]]))/Rx) - (1/Px);
\[Eta] = (0.05*(N[Log[2]]))/(V*Ry);
\[Rho] = ((Cy*(N[Log[2]]))/Ry) + (1/Py);
\[Phi] = kx*\[Psi];
\[Xi] = 0.05/V;
\[Kappa] = 1 - 0.05*h;
\[Mu] = (0.05*(N[Log[2]]))/(V*Ru);
\[Sigma] = ((Cu*(N[Log[2]]))/Ru) + (1/Py);

r1 = Plot[Evaluate[NDSolveValue[{
    x'[t] == x[t]*(\[Alpha] - a1*x[t] - a1*z[t] - (\[Beta]/h)*u[t]),
    y'[t] == y[t]*(\[Eta]*w[t] - \[Rho] - a2*y[t] - a2*u[t]),
    z'[t] == z[t]*(\[Alpha] - a1*x[t] - a1*z[t]) + (\[Beta]/h)*x[t]*u[t],
    u'[t] == u[t]*(\[Mu]*w[t] - \[Sigma] - a2*y[t] - a2*u[t] - \[Beta]*x[t]) + \[Chi]*z[t],
    w'[t] == \[Phi]*x[t] + \[Kappa]*\[Phi]*z[t] - \[Xi]*w[t]*y[t] - \[Xi]*w[t]*u[t],
    x[0] == 5.82, y[0] == 22.43, z[0] == 0.01, u[0] == 0.01,
    w[0] == 4152}, {x[t], y[t], z[t], u[t], (w[t]/100)}, {t, 0, 100}]], {t,
   0, 100}, PlotStyle -> Directive[Thickness[0.008], 12],
  PlotRange -> {{0, 30}, {0, 50}}, Frame -> True,
  FrameStyle -> Directive[Black, Thickness[0.006], 18],
```

```
  ImageSize -> {400, 300}, AspectRatio -> 0.75];
```

ky = 0.1

```
\[Alpha] = (((kx*\[Psi] - Cx)*(N[Log[2]]))/Rx) - (1/Px);
\[Eta] = (0.1*(N[Log[2]]))/(V*Ry);
\[Rho] = ((Cy*(N[Log[2]]))/Ry) + (1/Py);
\[Phi] = kx*\[Psi];
\[Xi] = 0.1/V;
\[Kappa] = 1 - 0.1*h;
\[Mu] = (0.1*(N[Log[2]]))/(V*Ru);
\[Sigma] = ((Cu*(N[Log[2]]))/Ru) + (1/Py);

r2 = Plot[Evaluate[NDSolveValue[{
    x'[t] == x[t]*(\[Alpha] - a1*x[t] - a1*z[t] - (\[Beta]/h)*u[t]),
    y'[t] == y[t]*(\[Eta]*w[t] - \[Rho] - a2*y[t] - a2*u[t]),
    z'[t] == z[t]*(\[Alpha] - a1*x[t] - a1*z[t]) + (\[Beta]/h)*x[t]*u[t],
    u'[t] == u[t]*(\[Mu]*w[t] - \[Sigma] - a2*y[t] - a2*u[t] - \[Beta]*x[t]) + \[Chi]*z[t],
    w'[t] == \[Phi]*x[t] + \[Kappa]*\[Phi]*z[t] - \[Xi]*w[t]*y[t] - \[Xi]* w[t]*u[t],
    x[0] == 5.82, y[0] == 22.43, z[0] == 0.01, u[0] == 0.01,
    w[0] == 2076}, {x[t], y[t], z[t], u[t], (w[t]/100)}, {t, 0, 100}]], {t,
   0, 100}, PlotStyle -> Directive[Thickness[0.008], 12],
  PlotRange -> {{0, 30}, {0, 50}}, Frame -> True,
  FrameStyle -> Directive[Black, Thickness[0.006], 18],
  ImageSize -> {400, 300}, AspectRatio -> 0.75];
```

ky = 0.15

```
\[Alpha] = (((kx*\[Psi] - Cx)*(N[Log[2]]))/Rx) - (1/Px);
\[Eta] = (0.15*(N[Log[2]]))/(V*Ry);
\[Rho] = ((Cy*(N[Log[2]]))/Ry) + (1/Py);
\[Phi] = kx*\[Psi];
```

```
\[Xi] = 0.15/V;
\[Kappa] = 1 - 0.15*h;
\[Mu] = (0.15*(N[Log[2]]))/(V*Ru);
\[Sigma] = ((Cu*(N[Log[2]]))/Ru) + (1/Py);

r3 = Plot[Evaluate[NDSolveValue[{
     x'[t] == x[t]*(\[Alpha] - a1*x[t] - a1*z[t] - (\[Beta]/h)*u[t]),
     y'[t] == y[t]*(\[Eta]*w[t] - \[Rho] - a2*y[t] - a2*u[t]),
     z'[t] == z[t]*(\[Alpha] - a1*x[t] - a1*z[t]) + (\[Beta]/h)*x[t]*u[t],
     u'[t] == u[t]*(\[Mu]*w[t] - \[Sigma] - a2*y[t] - a2*u[t] - \[Beta]*x[t]) + \[Chi]*z[t],
     w'[t] == \[Phi]*x[t] + \[Kappa]*\[Phi]*z[t] - \[Xi]*w[t]*y[t] - \[Xi]* w[t]*u[t],
     x[0] == 5.82, y[0] == 22.43, z[0] == 0.01, u[0] == 0.01,
     w[0] == 1384}, {x[t], y[t], z[t], u[t], (w[t]/100)}, {t, 0, 100}]], {t,
   0, 100}, PlotStyle -> Directive[Thickness[0.008], 12],
  PlotRange -> {{0, 30}, {0, 50}}, Frame -> True,
  FrameStyle -> Directive[Black, Thickness[0.006], 18],
  ImageSize -> {400, 300}, AspectRatio -> 0.75];
```

ky = 0.2

```
\[Alpha] = (((kx*\[Psi] - Cx)*(N[Log[2]]))/Rx) - (1/Px);
\[Eta] = (0.2*(N[Log[2]]))/(V*Ry);
\[Rho] = ((Cy*(N[Log[2]]))/Ry) + (1/Py);
\[Phi] = kx*\[Psi];
\[Xi] = 0.2/V;
\[Kappa] = 1 - 0.2*h;
\[Mu] = (0.2*(N[Log[2]]))/(V*Ru);
\[Sigma] = ((Cu*(N[Log[2]]))/Ru) + (1/Py);

r4 = Plot[Evaluate[NDSolveValue[{
     x'[t] == x[t]*(\[Alpha] - a1*x[t] - a1*z[t] - (\[Beta]/h)*u[t]),
     y'[t] == y[t]*(\[Eta]*w[t] - \[Rho] - a2*y[t] - a2*u[t]),
```

```
    z'[t] == z[t]*(\[Alpha] - a1*x[t] - a1*z[t]) + (\[Beta]/h)*x[t]*u[t],
    u'[t] == u[t]*(\[Mu]*w[t] - \[Sigma] - a2*y[t] -  a2*u[t] - \[Beta]*x[t]) + \[Chi]*z[t],
    w'[t] == \[Phi]*x[t] + \[Kappa]*\[Phi]*z[t] - \[Xi]*w[t]*y[t] - \[Xi]* w[t]*u[t],
    x[0] == 5.82, y[0] == 22.43, z[0] == 0.01, u[0] == 0.01,
    w[0] == 1038}, {x[t], y[t], z[t], u[t], (w[t]/100)}, {t, 0, 100}]], {t,
  0, 100}, PlotStyle -> Directive[Thickness[0.008], 12],
 PlotRange -> {{0, 30}, {0, 50}}, Frame -> True,
 FrameStyle -> Directive[Black, Thickness[0.006], 18],
 ImageSize -> {400, 300}, AspectRatio -> 0.75];
```

Combined

```
Figureky =
 ResourceFunction["PlotGrid"][{{r1}, {r2}, {r3}, {r4}}, Spacings -> 40,
  PlotLabel ->
   Style["(b) \!\(\*SubscriptBox[\(k\), \(Y\)]\)", FontFamily -> "Arial", 36,
    Bold, Black],
  FrameStyle -> Directive[Black, FontFamily -> "Arial", FontSize -> 40],
  PlotLabels -> {
    Placed[ Framed[Style["\!\(\*SubscriptBox[\(k\), \(Y\)]\) = 0.05",
      FontFamily -> "Arial", 24, Bold, Black]], {Right, Top}],
    Placed[Framed[Style["\!\(\*SubscriptBox[\(k\), \(Y\)]\) = 0.1",
      FontFamily -> "Arial", 24, Bold, Black],
     Background -> LightGray], {Right, Top}],
    Placed[Framed[ Style["\!\(\*SubscriptBox[\(k\), \(Y\)]\) = 0.15",
      FontFamily -> "Arial", 24, Bold, Black]], {Right, Top}],
    Placed[Framed[
     Style["\!\(\*SubscriptBox[\(k\), \(Y\)]\) = 0.2",
      FontFamily -> "Arial", 24, Bold, Black]], {Right, Top}] }
  , ImageSize -> {400, 1200}]
```

Impact of interaction rate, \[Beta] (Figure 5c)

```
ClearAll["Global`*"];

a1 = 3; a2 = 2; \[Psi] = 400; kx = 0.2; Cx = 4; Rx = 3; Px = 10; ky = 0.1; Ry = 0.3; V = 10; Cy =
1.2; Py = 3; \[Chi] = 30; Ru = 0.4; Cu = 1.3; h = 4;

\[Alpha] = (((kx*\[Psi] - Cx)*(N[Log[2]]))/Rx) - (1/Px);
\[Eta] = (ky*(N[Log[2]]))/(V*Ry);
\[Rho] = ((Cy*(N[Log[2]]))/Ry) + (1/Py);
\[Phi] = kx*\[Psi];
\[Xi] = ky/V;
\[Kappa] = 1 - ky*h;
\[Mu] = (ky*(N[Log[2]]))/(V*Ru);
\[Sigma] = ((Cu*(N[Log[2]]))/Ru) + (1/Py);

\[Beta] = 0.1

r1 = Plot[Evaluate[NDSolveValue[{
    x'[t] == x[t]*(\[Alpha] - a1*x[t] - a1*z[t] - (0.1/h)*u[t]),
    y'[t] == y[t]*(\[Eta]*w[t] - \[Rho] - a2*y[t] - a2*u[t]),
    z'[t] == z[t]*(\[Alpha] - a1*x[t] - a1*z[t]) + (0.1/h)*x[t]*u[t],
    u'[t] == u[t]*(\[Mu]*w[t] - \[Sigma] - a2*y[t] - a2*u[t] - 0.1*x[t]) + \[Chi]* z[t],
    w'[t] == \[Phi]*x[t] + \[Kappa]*\[Phi]*z[t] - \[Xi]*w[t]*y[t] - \[Xi]*w[t]*u[t],
    x[0] == 5.82, y[0] == 22.43, z[0] == 0.01, u[0] == 0.01,
    w[0] == 2076}, {x[t], y[t], z[t], u[t], (w[t]/100)}, {t, 0, 100}]], {t,
   0, 100}, PlotStyle -> Directive[Thickness[0.008], 12],
  PlotRange -> {{0, 50}, {0, 30}}, Frame -> True,
  FrameStyle -> Directive[Black, Thickness[0.006], 18],
  ImageSize -> {400, 300}, AspectRatio -> 0.75];

\[Beta] = 0.5
```

```
r2 = Plot[Evaluate[NDSolveValue[{
    x'[t] == x[t]*(\[Alpha] - a1*x[t] - a1*z[t] - (0.5/h)*u[t]),
    y'[t] == y[t]*(\[Eta]*w[t] - \[Rho] - a2*y[t] - a2*u[t]),
    z'[t] == z[t]*(\[Alpha] - a1*x[t] - a1*z[t]) + (0.5/h)*x[t]*u[t],
    u'[t] == u[t]*(\[Mu]*w[t] - \[Sigma] - a2*y[t] - a2*u[t] - 0.5*x[t]) + \[Chi]*z[t],
    w'[t] == \[Phi]*x[t] + \[Kappa]*\[Phi]*z[t] - \[Xi]*w[t]*y[t] - \[Xi]*w[t]*u[t],
    x[0] == 5.82, y[0] == 22.43, z[0] == 0.01, u[0] == 0.01,
    w[0] == 2076}, {x[t], y[t], z[t], u[t], (w[t]/100)}, {t, 0, 100}]], {t,
   0, 100}, PlotStyle -> Directive[Thickness[0.008], 12],
  PlotRange -> {{0, 50}, {0, 30}}, Frame -> True,
  FrameStyle -> Directive[Black, Thickness[0.006], 18],
  ImageSize -> {400, 300}, AspectRatio -> 0.75];

\[Beta] = 0.8

r3 = Plot[Evaluate[NDSolveValue[{
    x'[t] == x[t]*(\[Alpha] - a1*x[t] - a1*z[t] - (0.8/h)*u[t]),
    y'[t] == y[t]*(\[Eta]*w[t] - \[Rho] - a2*y[t] - a2*u[t]),
    z'[t] == z[t]*(\[Alpha] - a1*x[t] - a1*z[t]) + (0.8/h)*x[t]*u[t],
    u'[t] == u[t]*(\[Mu]*w[t] - \[Sigma] - a2*y[t] - a2*u[t] - 0.8*x[t]) + \[Chi]*z[t],
    w'[t] == \[Phi]*x[t] + \[Kappa]*\[Phi]*z[t] - \[Xi]*w[t]*y[t] - \[Xi]*w[t]*u[t],
    x[0] == 5.82, y[0] == 22.43, z[0] == 0.01, u[0] == 0.01,
    w[0] == 2076}, {x[t], y[t], z[t], u[t], (w[t]/100)}, {t, 0, 100}]], {t,
   0, 100}, PlotStyle -> Directive[Thickness[0.008], 12],
  PlotRange -> {{0, 50}, {0, 30}}, Frame -> True,
  FrameStyle -> Directive[Black, Thickness[0.006], 18],
  ImageSize -> {400, 300}, AspectRatio -> 0.75];

\[Beta] = 0.99

r4 = Plot[Evaluate[NDSolveValue[{
```

```
    x'[t] == x[t]*(\[Alpha] - a1*x[t] - a1*z[t] - (0.99/h)*u[t]),
    y'[t] == y[t]*(\[Eta]*w[t] - \[Rho] - a2*y[t] - a2*u[t]),
    z'[t] == z[t]*(\[Alpha] - a1*x[t] - a1*z[t]) + (0.99/h)*x[t]*u[t],
    u'[t] == u[t]*(\[Mu]*w[t] - \[Sigma] - a2*y[t] - a2*u[t] - 0.99*x[t]) + \[Chi]* z[t],
    w'[t] == \[Phi]*x[t] + \[Kappa]*\[Phi]*z[t] - \[Xi]*w[t]*y[t] - \[Xi]* w[t]*u[t],
    x[0] == 5.82, y[0] == 22.43, z[0] == 0.01, u[0] == 0.01,
    w[0] == 2076}, {x[t], y[t], z[t], u[t], (w[t]/100)}, {t, 0, 100}]], {t,
   0, 100}, PlotStyle -> Directive[Thickness[0.008], 12],
  PlotRange -> {{0, 50}, {0, 30}}, Frame -> True,
  FrameStyle -> Directive[Black, Thickness[0.006], 18],
  ImageSize -> {400, 300}, AspectRatio -> 0.75];
```

Combined

```
Figurebeta =
 ResourceFunction["PlotGrid"][{{r1}, {r2}, {r3}, {r4}}, Spacings -> 40,
  PlotLabel -> Style["(c) \[Beta]", FontFamily -> "Arial", 36, Bold, Black],
  FrameLabel -> {{None, None}, {None, None}},
  FrameStyle -> Directive[Black, FontFamily -> "Arial", FontSize -> 40],
  PlotLabels -> {Placed[
     Framed[Style["\[Beta] = 0.1", FontFamily -> "Arial", 24, Bold,
       Black]], {Right, Top}],
    Placed[Framed[
      Style["\[Beta] = 0.5", FontFamily -> "Arial", 24, Bold,
       Black]], {Right, Top}],
    Placed[Framed[
      Style["\[Beta] = 0.8", FontFamily -> "Arial", 24, Bold, Black],
      Background -> LightGray], {Right, Top}],
    Placed[Framed[
      Style["\[Beta] = 0.99", FontFamily -> "Arial", 24, Bold,
       Black]], {Right, Top}]
    }
```

```
, ImageSize -> {400, 1200}]
```

Impact of host food, \[Psi] (Figure 5d)

```
ClearAll["Global`*"];

a1 = 3; a2 = 2; kx = 0.2; V = 10; Cx = 4; Rx = 3; Px = 10; ky = 0.1; Ry = 0.3; Cy = 1.2; Py = 3;
\[Beta] = 0.8; h = 4; \[Chi] = 30; Ru = 0.4; Cu = 1.3;
```

\[Psi] = 200

```
\[Alpha] = (((kx*(200) - Cx)*(N[Log[2]]))/Rx) - (1/Px);
\[Eta] = (ky*(N[Log[2]]))/(V*Ry);
\[Rho] = ((Cy*(N[Log[2]]))/Ry) + (1/Py);
\[Phi] = kx*(200);
\[Xi] = ky/V;
\[Kappa] = 1 - ky*h;
\[Mu] = (ky*(N[Log[2]]))/(V*Ru);
\[Sigma] = ((Cu*(N[Log[2]]))/Ru) + (1/Py);

r1 = Plot[Evaluate[NDSolveValue[{
    x'[t] == x[t]*(\[Alpha] - a1*x[t] - a1*z[t] - (\[Beta]/h)*u[t]),
    y'[t] == y[t]*(\[Eta]*w[t] - \[Rho] - a2*y[t] - a2*u[t]),
    z'[t] == z[t]*(\[Alpha] - a1*x[t] - a1*z[t]) + (\[Beta]/h)*x[t]*u[t],
    u'[t] == u[t]*(\[Mu]*w[t] - \[Sigma] - a2*y[t] - a2*u[t] - \[Beta]*x[t]) + \[Chi]*z[t],
    w'[t] == \[Phi]*x[t] + \[Kappa]*\[Phi]*z[t] - \[Xi]*w[t]*y[t] - \[Xi]*w[t]*u[t],
    x[0] == 2.74, y[0] == 10.50, z[0] == 0.01, u[0] == 0.01,
    w[0] == 1043}, {x[t], y[t], z[t], u[t], (w[t]/100)}, {t, 0, 100}]], {t,
  0, 100}, PlotStyle -> Directive[Thickness[0.008], 12],
 PlotRange -> {{0, 30}, {0, 60}}, Frame -> True,
 FrameStyle -> Directive[Black, Thickness[0.006], 18],
 ImageSize -> {400, 300}, AspectRatio -> 0.75];
```

\[Psi] = 400

```
\[Alpha] = (((kx*(400) - Cx)*(N[Log[2]]))/Rx) - (1/Px);
\[Eta] = (ky*(N[Log[2]]))/(V*Ry);
\[Rho] = ((Cy*(N[Log[2]]))/Ry) + (1/Py);
\[Phi] = kx*(400);
\[Xi] = ky/V;
\[Kappa] = 1 - ky*h;
\[Mu] = (ky*(N[Log[2]]))/(V*Ru);
\[Sigma] = ((Cu*(N[Log[2]]))/Ru) + (1/Py);

r2 = Plot[Evaluate[NDSolveValue[{
    x'[t] == x[t]*(\[Alpha] - a1*x[t] - a1*z[t] - (\[Beta]/h)*u[t]),
    y'[t] == y[t]*(\[Eta]*w[t] - \[Rho] - a2*y[t] - a2*u[t]),
    z'[t] == z[t]*(\[Alpha] - a1*x[t] - a1*z[t]) + (\[Beta]/h)*x[t]*u[t],
    u'[t] == u[t]*(\[Mu]*w[t] - \[Sigma] - a2*y[t] - a2*u[t] - \[Beta]*x[t]) + \[Chi]*z[t],
    w'[t] == \[Phi]*x[t] + \[Kappa]*\[Phi]*z[t] - \[Xi]*w[t]*y[t] - \[Xi]*w[t]*u[t],
    x[0] == 5.82, y[0] == 22.43, z[0] == 0.01, u[0] == 0.01,
    w[0] == 2076}, {x[t], y[t], z[t], u[t], (w[t]/100)}, {t, 0, 100}]], {t,
   0, 100}, PlotStyle -> Directive[Thickness[0.008], 12],
  PlotRange -> {{0, 30}, {0, 60}}, Frame -> True,
  FrameStyle -> Directive[Black, Thickness[0.006], 18],
  ImageSize -> {400, 300}, AspectRatio -> 0.75];
```

\[Psi] = 600

```
\[Alpha] = (((kx*(600) - Cx)*(N[Log[2]]))/Rx) - (1/Px);
\[Eta] = (ky*(N[Log[2]]))/(V*Ry);
\[Rho] = ((Cy*(N[Log[2]]))/Ry) + (1/Py);
\[Phi] = kx*(600);
\[Xi] = ky/V;
```

```
\[Kappa] = 1 - ky*h;
\[Mu] = (ky*(N[Log[2]]))/(V*Ru);
\[Sigma] = ((Cu*(N[Log[2]]))/Ru) + (1/Py);

r3 = Plot[Evaluate[NDSolveValue[{
    x'[t] == x[t]*(\[Alpha] - a1*x[t] - a1*z[t] - (\[Beta]/h)*u[t]),
    y'[t] == y[t]*(\[Eta]*w[t] - \[Rho] - a2*y[t] - a2*u[t]),
    z'[t] == z[t]*(\[Alpha] - a1*x[t] - a1*z[t]) + (\[Beta]/h)*x[t]*u[t],
    u'[t] == u[t]*(\[Mu]*w[t] - \[Sigma] - a2*y[t] -  a2*u[t] - \[Beta]*x[t]) + \[Chi]*z[t],
    w'[t] == \[Phi]*x[t] + \[Kappa]*\[Phi]*z[t] - \[Xi]*w[t]*y[t] - \[Xi]*w[t]*u[t],
    x[0] == 8.90, y[0] == 34.36, z[0] == 0.01, u[0] == 0.01,
    w[0] == 3109}, {x[t], y[t], z[t], u[t], (w[t]/100)}, {t, 0, 100}]], {t,
  0, 100}, PlotStyle -> Directive[Thickness[0.008], 12],
 PlotRange -> {{0, 30}, {0, 60}}, Frame -> True,
 FrameStyle -> Directive[Black, Thickness[0.006], 18],
 ImageSize -> {400, 300}, AspectRatio -> 0.75];
```

\[Psi] = 1000

```
\[Alpha] = (((kx*(1000) - Cx)*(N[Log[2]]))/Rx) - (1/Px);
\[Eta] = (ky*(N[Log[2]]))/(V*Ry);
\[Rho] = ((Cy*(N[Log[2]]))/Ry) + (1/Py);
\[Phi] = kx*(1000);
\[Xi] = ky/V;
\[Kappa] = 1 - ky*h;
\[Mu] = (ky*(N[Log[2]]))/(V*Ru);
\[Sigma] = ((Cu*(N[Log[2]]))/Ru) + (1/Py);

r4 = Plot[Evaluate[NDSolveValue[{
    x'[t] == x[t]*(\[Alpha] - a1*x[t] - a1*z[t] - (\[Beta]/h)*u[t]),
    y'[t] == y[t]*(\[Eta]*w[t] - \[Rho] - a2*y[t] - a2*u[t]),
    z'[t] == z[t]*(\[Alpha] - a1*x[t] - a1*z[t]) + (\[Beta]/h)*x[t]*u[t],
```

```
    u'[t] == u[t]*(\[Mu]*w[t] - \[Sigma] - a2*y[t] - a2*u[t] - \[Beta]*x[t]) + \[Chi]*z[t],
    w'[t] == \[Phi]*x[t] + \[Kappa]*\[Phi]*z[t] - \[Xi]*w[t]*y[t] - \[Xi]* w[t]*u[t],
    x[0] == 15.06, y[0] == 58.22, z[0] == 0.01, u[0] == 0.01,
    w[0] == 5174}, {x[t], y[t], z[t], u[t], (w[t]/100)}, {t, 0, 100}]], {t,
   0, 100}, PlotStyle -> Directive[Thickness[0.008], 12],
  PlotRange -> {{0, 30}, {0, 60}}, Frame -> True,
  FrameStyle -> Directive[Black, Thickness[0.006], 18],
  ImageSize -> {400, 300}, AspectRatio -> 0.75];
```

Combined

```
Figurepsi =
 ResourceFunction["PlotGrid"][{{r1}, {r2}, {r3}, {r4}}, Spacings -> 40,
  PlotLabel -> Style["(d) \[Psi]", FontFamily -> "Arial", 36, Bold, Black],
  FrameStyle -> Directive[Black, FontFamily -> "Arial", FontSize -> 40],
  PlotLabels -> {Placed[ Framed[Style["\[Psi] = 200", FontFamily -> "Arial", 24, Bold,
      Black]], {Right, Top}],
    Placed[Framed[
     Style["\[Psi] = 400", FontFamily -> "Arial", 24, Bold, Black],
     Background -> LightGray], {Right, Top}],
    Placed[Framed[
     Style["\[Psi] = 600", FontFamily -> "Arial", 24, Bold, Black]], {Right,
      Top}], Placed[
    Framed[Style["\[Psi] = 1000", FontFamily -> "Arial", 24, Bold,
      Black]], {Right, Top}]
    }
  , ImageSize -> {400, 1200}]
```

Impact of no. of ectosymbionts, h (Figure 5e)

```
ClearAll["Global`*"];
```

```
a1 = 3; a2 = 2; \[Psi] = 400; kx = 0.2; Cx = 4; Rx = 3; Px = 10; ky = 0.1; Ry = 0.3; V = 10; Cy =
1.2; Py = 3; \[Beta] = 0.8; \[Chi] = 30; Ru = 0.4; Cu = 1.3;

\[Alpha] = (((kx*\[Psi] - Cx)*(N[Log[2]]))/Rx) - (1/Px);
\[Eta] = (ky*(N[Log[2]]))/(V*Ry);
\[Rho] = ((Cy*(N[Log[2]]))/Ry) + (1/Py);
\[Phi] = kx*\[Psi];
\[Xi] = ky/V;
\[Mu] = (ky*(N[Log[2]]))/(V*Ru);
\[Sigma] = ((Cu*(N[Log[2]]))/Ru) + (1/Py);

h = 1

r1 = Plot[Evaluate[NDSolveValue[{
    x'[t] == x[t]*(\[Alpha] - a1*x[t] - a1*z[t] - (\[Beta]/1)*u[t]),
    y'[t] == y[t]*(\[Eta]*w[t] - \[Rho] - a2*y[t] - a2*u[t]),
    z'[t] == z[t]*(\[Alpha] - a1*x[t] - a1*z[t]) + (\[Beta]/1)*x[t]*u[t],
    u'[t] ==  u[t]*(\[Mu]*w[t] - \[Sigma] - a2*y[t] - a2*u[t] - \[Beta]*x[t]) + \[Chi]*z[t],
    w'[t] == \[Phi]*x[t] + (1 - ky*1)*\[Phi]*z[t] - \[Xi]*w[t]*y[t] - \[Xi]* w[t]*u[t],
    x[0] == 5.82, y[0] == 22.43, z[0] == 0.01, u[0] == 0.01,
    w[0] == 2076}, {x[t], y[t], z[t], u[t], (w[t]/100)}, {t, 0, 100}]], {t,
  0, 100}, PlotStyle -> Directive[Thickness[0.008], 12],
  PlotRange -> {{0, 50}, {0, 30}}, Frame -> True,
  FrameStyle -> Directive[Black, Thickness[0.006], 18],
  ImageSize -> {400, 300}, AspectRatio -> 0.75];

h = 2

r2 = Plot[Evaluate[NDSolveValue[{
    x'[t] == x[t]*(\[Alpha] - a1*x[t] - a1*z[t] - (\[Beta]/2)*u[t]),
    y'[t] == y[t]*(\[Eta]*w[t] - \[Rho] - a2*y[t] - a2*u[t]),
```

```
    z'[t] == z[t]*(\[Alpha] - a1*x[t] - a1*z[t]) + (\[Beta]/2)*x[t]*u[t],
    u'[t] == u[t]*(\[Mu]*w[t] - \[Sigma] - a2*y[t] - a2*u[t] - \[Beta]*x[t]) + \[Chi]*z[t],
    w'[t] == \[Phi]*x[t] + (1 - ky*2)*\[Phi]*z[t] - \[Xi]*w[t]*y[t] - \[Xi]*w[t]*u[t],
    x[0] == 5.82, y[0] == 22.43, z[0] == 0.01, u[0] == 0.01,
    w[0] == 2076}, {x[t], y[t], z[t], u[t], (w[t]/100)}, {t, 0, 100}]], {t,
   0, 100}, PlotStyle -> Directive[Thickness[0.008], 12],
  PlotRange -> {{0, 50}, {0, 30}}, Frame -> True,
  FrameStyle -> Directive[Black, Thickness[0.006], 18],
  ImageSize -> {400, 300}, AspectRatio -> 0.75];

h = 4

r3 = Plot[Evaluate[NDSolveValue[{
    x'[t] == x[t]*(\[Alpha] - a1*x[t] - a1*z[t] - (\[Beta]/4)*u[t]),
    y'[t] == y[t]*(\[Eta]*w[t] - \[Rho] - a2*y[t] - a2*u[t]),
    z'[t] == z[t]*(\[Alpha] - a1*x[t] - a1*z[t]) + (\[Beta]/4)*x[t]*u[t],
    u'[t] == u[t]*(\[Mu]*w[t] - \[Sigma] - a2*y[t] - a2*u[t] - \[Beta]*x[t]) + \[Chi]*z[t],
    w'[t] == \[Phi]*x[t] + (1 - ky*4)*\[Phi]*z[t] - \[Xi]*w[t]*y[t] - \[Xi]*w[t]*u[t],
    x[0] == 5.82, y[0] == 22.43, z[0] == 0.01, u[0] == 0.01,
    w[0] == 2076}, {x[t], y[t], z[t], u[t], (w[t]/100)}, {t, 0, 100}]], {t,
   0, 100}, PlotStyle -> Directive[Thickness[0.008], 12],
  PlotRange -> {{0, 50}, {0, 30}}, Frame -> True,
  FrameStyle -> Directive[Black, Thickness[0.006], 18],
  ImageSize -> {400, 300}, AspectRatio -> 0.75];

h = 8

r4 = Plot[Evaluate[NDSolveValue[{
    x'[t] == x[t]*(\[Alpha] - a1*x[t] - a1*z[t] - (\[Beta]/8)*u[t]),
    y'[t] == y[t]*(\[Eta]*w[t] - \[Rho] - a2*y[t] - a2*u[t]),
    z'[t] == z[t]*(\[Alpha] - a1*x[t] - a1*z[t]) + (\[Beta]/8)*x[t]*u[t],
    u'[t] == u[t]*(\[Mu]*w[t] - \[Sigma] - a2*y[t] - a2*u[t] - \[Beta]*x[t]) + \[Chi]*z[t],
```

```
    w'[t] == \[Phi]*x[t] + (1 - ky*8)*\[Phi]*z[t] - \[Xi]*w[t]*y[t] - \[Xi]*w[t]*u[t],
    x[0] == 5.82, y[0] == 22.43, z[0] == 0.01, u[0] == 0.01,
    w[0] == 2076}, {x[t], y[t], z[t], u[t], (w[t]/100)}, {t, 0, 100}]], {t,
   0, 100}, PlotStyle -> Directive[Thickness[0.008], 12],
  PlotRange -> {{0, 50}, {0, 30}}, Frame -> True,
  FrameStyle -> Directive[Black, Thickness[0.006], 18],
  ImageSize -> {400, 300}, AspectRatio -> 0.75];
```

Combined

```
Figureh =
  ResourceFunction["PlotGrid"][{{r1}, {r2}, {r3}, {r4}}, Spacings -> 40,
  PlotLabel -> Style["(e) \!\(\*
StyleBox[\"h\",\nFontSlant->\"Italic\"]\)", FontFamily -> "Arial", 36, Bold,
    Black], FrameStyle ->
   Directive[Black, FontFamily -> "Arial", FontSize -> 40],
  PlotLabels -> {Placed[Framed[Style["\!\(\*
StyleBox[\"h\",\nFontSlant->\"Italic\"]\) = 1", FontFamily -> "Arial", 24,
       Bold, Black]], {Right, Top}], Placed[Framed[Style["\!\(\*
StyleBox[\"h\",\nFontSlant->\"Italic\"]\) = 2", FontFamily -> "Arial", 24,
       Bold, Black]], {Right, Top}], Placed[Framed[Style["\!\(\*
StyleBox[\"h\",\nFontSlant->\"Italic\"]\) = 4", FontFamily -> "Arial", 24,
       Bold, Black], Background -> LightGray], {Right, Top}],
    Placed[Framed[Style["\!\(\*
StyleBox[\"h\",\nFontSlant->\"Italic\"]\) = 8", FontFamily -> "Arial", 24,
       Bold, Black]], {Right, Top}]
    }
  , ImageSize -> {400, 1200}]
```

Vertical transmission (Figure 6)

```
ClearAll["Global`*"];

a1 = 3; a2 = 2; \[Psi] = 400; kx = 0.2; Cx = 4; Rx = 3; Px = 10; ky = 0.1; Ry = 0.3; V = 10; Cy =
1.2; Py = 3; \[Beta] = 0.8; h = 4; \[Chi] = 30; Ru = 0.4; Cu = 1.3;

\[Alpha] = (((kx*\[Psi] - Cx)*(N[Log[2]]))/Rx) - (1/Px);
\[Eta] = (ky*(N[Log[2]]))/(V*Ry);
\[Rho] = ((Cy*(N[Log[2]]))/Ry) + (1/Py);
\[Phi] = kx*\[Psi];
\[Xi] = ky/V;
\[Kappa] = 1 - ky*h;
\[Mu] = (ky*(N[Log[2]]))/(V*Ru);
\[Sigma] = ((Cu*(N[Log[2]]))/Ru) + (1/Py);

\[Theta] = 1

r1 = Plot[Evaluate[NDSolveValue[{
    x'[t] == x[t]*(\[Alpha] - a1*x[t] - a1*z[t] - (\[Beta]/h)*u[t]) + (1 - 1)*\[Alpha]*z[t],
    y'[t] == y[t]*(\[Eta]*w[t] - \[Rho] - a2*y[t] - a2*u[t]),
    z'[t] == z[t]*(1*\[Alpha] - a1*x[t] - a1*z[t]) + (\[Beta]/h)*x[t]*u[t],
    u'[t] == u[t]*(\[Mu]*w[t] - \[Sigma] - a2*y[t] - a2*u[t] - \[Beta]*x[t]) + \[Chi]*z[t],
    w'[t] == \[Phi]*x[t] + \[Kappa]*\[Phi]*z[t] - \[Xi]*w[t]*y[t] - \[Xi]*w[t]*u[t],
    x[0] == 5.82, y[0] == 22.43, z[0] == 0.01, u[0] == 0.01,
    w[0] == 2076}, {x[t], y[t], z[t], u[t], (w[t]/100)}, {t, 0, 100}]], {t,
   0, 100}, PlotStyle -> Directive[Thickness[0.008], 12],
  PlotRange -> {{0, 25}, {0, 30}},
  PlotLegends -> Placed[LineLegend[Automatic, {Style["Free-living host, \!\(\*
StyleBox[\"x\",\nFontSlant->\"Italic\"]\)", FontFamily -> "Arial", 14, Black],
     Style["Free-living symbiont, \!\(\*
StyleBox[\"y\",\nFontSlant->\"Italic\"]\)", FontFamily -> "Arial", 14, Black],
```

```
        Style["Consortia, \!\(\*
StyleBox[\"z\",\nFontSlant->\"Italic\"]\)", FontFamily -> "Arial", 14, Black],
        Style["Free-living mutant symbiont, \!\(\*
StyleBox[\"u\",\nFontSlant->\"Italic\"]\)", FontFamily -> "Arial", 14, Black],
        Style["Metabolite concentration, \!\(\*
StyleBox[\"w\",\nFontSlant->\"Italic\"]\)/100", FontFamily -> "Arial", 14,
        Black]}, LegendLayout -> (Grid[#, Spacings -> {.5, .25},
          Alignment -> Left] &),
      LegendFunction -> (Framed[#, Background -> White, FrameMargins -> 1.0,
          FrameStyle -> AbsoluteThickness[0.5],
          RoundingRadius -> 0] &)], {{0.65, 0.98}, {0.5, 1.0}}],
   Frame -> True, FrameStyle -> Directive[Black, Thickness[0.006], 18],
   ImageSize -> {400, 300}, AspectRatio -> 0.75];

\[Theta] = 0.99

r2 = Plot[Evaluate[NDSolveValue[{
      x'[t] ==  x[t]*(\[Alpha] - a1*x[t] - a1*z[t] - (\[Beta]/h)*u[t]) + (1 -  0.99)*\[Alpha]*z[t],
      y'[t] == y[t]*(\[Eta]*w[t] - \[Rho] - a2*y[t] - a2*u[t]),
      z'[t] ==  z[t]*(0.99*\[Alpha] - a1*x[t] - a1*z[t]) + (\[Beta]/h)*x[t]*u[t],
      u'[t] == u[t]*(\[Mu]*w[t] - \[Sigma] - a2*y[t] -  a2*u[t] - \[Beta]*x[t]) + \[Chi]*z[t],
      w'[t] == \[Phi]*x[t] + \[Kappa]*\[Phi]*z[t] - \[Xi]*w[t]*y[t] - \[Xi]*w[t]*u[t],
      x[0] == 5.82, y[0] == 22.43, z[0] == 0.01, u[0] == 0.01,
      w[0] == 2076}, {x[t], y[t], z[t], u[t], (w[t]/100)}, {t, 0, 100}]], {t,
    0, 100}, PlotStyle -> Directive[Thickness[0.008], 12],
   PlotRange -> {{0, 25}, {0, 30}}, Frame -> True,
   FrameStyle -> Directive[Black, Thickness[0.006], 18],
   ImageSize -> {400, 300}, AspectRatio -> 0.75];

\[Theta] = 0.75

r3 = Plot[Evaluate[NDSolveValue[{
```

```
    x'[t] == x[t]*(\[Alpha] - a1*x[t] - a1*z[t] - (\[Beta]/h)*u[t]) + (1 - 0.75)*\[Alpha]*z[t],
    y'[t] == y[t]*(\[Eta]*w[t] - \[Rho] - a2*y[t] - a2*u[t]),
    z'[t] == z[t]*(0.75*\[Alpha] - a1*x[t] - a1*z[t]) + (\[Beta]/h)*x[t]*u[t],
    u'[t] == u[t]*(\[Mu]*w[t] - \[Sigma] - a2*y[t] -  a2*u[t] - \[Beta]*x[t]) + \[Chi]*z[t],
    w'[t] == \[Phi]*x[t] + \[Kappa]*\[Phi]*z[t] - \[Xi]*w[t]*y[t] - \[Xi]*w[t]*u[t],
    x[0] == 5.82, y[0] == 22.43, z[0] == 0.01, u[0] == 0.01,
    w[0] == 2076}, {x[t], y[t], z[t], u[t], (w[t]/100)}, {t, 0, 100}]], {t,
   0, 100}, PlotStyle -> Directive[Thickness[0.008], 12],
  PlotRange -> {{0, 25}, {0, 30}}, Frame -> True,
  FrameStyle -> Directive[Black, Thickness[0.006], 18],
  ImageSize -> {400, 300}, AspectRatio -> 0.75];
```

\[Theta] = 0.5

```
r4 = Plot[Evaluate[NDSolveValue[{
    x'[t] == x[t]*(\[Alpha] - a1*x[t] - a1*z[t] - (\[Beta]/h)*u[t]) + (1 - 0.5)*\[Alpha]*z[t],
    y'[t] == y[t]*(\[Eta]*w[t] - \[Rho] - a2*y[t] - a2*u[t]),
    z'[t] == z[t]*(0.5*\[Alpha] - a1*x[t] - a1*z[t]) + (\[Beta]/h)*x[t]*u[t],
    u'[t] == u[t]*(\[Mu]*w[t] - \[Sigma] - a2*y[t] - a2*u[t] - \[Beta]*x[t]) + \[Chi]*z[t],
    w'[t] == \[Phi]*x[t] + \[Kappa]*\[Phi]*z[t] - \[Xi]*w[t]*y[t] - \[Xi]*w[t]*u[t],
    x[0] == 5.82, y[0] == 22.43, z[0] == 0.01, u[0] == 0.01,
    w[0] == 2076}, {x[t], y[t], z[t], u[t], (w[t]/100)}, {t, 0, 100}]], {t,
   0, 100}, PlotStyle -> Directive[Thickness[0.008], 12],
  PlotRange -> {{0, 25}, {0, 30}}, Frame -> True,
  FrameStyle -> Directive[Black, Thickness[0.006], 18],
  ImageSize -> {400, 300}, AspectRatio -> 0.75];
```

Combined

```
Figuretheta =
 ResourceFunction["PlotGrid"][{{r4, r3, r2, r1}}, Spacings -> 50,
  FrameLabel -> {{Pane["Densities"], None}, {"Time", None}},
```

```
  FrameStyle -> Directive[Black, FontFamily -> "Arial", FontSize -> 32],
  PlotLabels -> {Placed[
    Framed[Style["\[Theta] = 0.5", FontFamily -> "Arial", 22, Bold,
      Black]], {Left, Top}],
   Placed[Framed[
     Style["\[Theta] = 0.75", FontFamily -> "Arial", 22, Bold,
      Black]], {Left, Top}],
   Placed[Framed[
     Style["\[Theta] = 0.99", FontFamily -> "Arial", 22, Bold,
      Black]], {Left, Top}],
   Placed[Framed[
     Style["\[Theta] = 1.0", FontFamily -> "Arial", 22, Bold, Black],
     Background -> LightGray], {Left, Top}]
   }
  , ImageSize -> {1750, 400}]
```

RegionPlot for ES (Figure S1)

```
ClearAll["Global`*"];

FigSI = RegionPlot[{1.2 < y < 4*x, y > 5.55*((x - 0.3)/(x)),
   1.2 < y < 4*x && y > 5.55*((x - 0.3)/(x))}, {x, 0, 1}, {y, 0, 2},
  FrameLabel -> {Style["\!\(\*SubscriptBox[
StyleBox[\"R\",\nFontSlant->\"Italic\"], \(U\)]\)", FontFamily -> "Arial",
    18], Style["\!\(\*SubscriptBox[
StyleBox[\"C\",\nFontSlant->\"Italic\"], \(U\)]\) & \!\(\*SuperscriptBox[\(\
\[Chi]\), \(*\)]\)", FontFamily -> "Arial", 18]},
  BoundaryStyle -> Directive[Thickness[0.006], Dashed],
  FrameStyle -> Directive[Black, Thickness[0.006], 14],
  PlotLegends -> Placed[SwatchLegend[Automatic, {Style["\!\(\*SubscriptBox[
StyleBox[\"C\",\nFontSlant->\"Italic\"], \(U\)]\)/\!\(\*SubscriptBox[
```

```
StyleBox[\"R\",\nFontSlant->\"Italic\"], \(U\)]\) < \!\(\*SubscriptBox[
StyleBox[\"C\",\nFontSlant->\"Italic\"], \(Y\)]\)/\!\(\*SubscriptBox[
StyleBox[\"R\",\nFontSlant->\"Italic\"], \(Y\)]\)", FontFamily -> "Arial", 14,
     Black], Style[
    " \!\(\*SuperscriptBox[\(\[Chi]\), \(*\)]\) > \!\(\*SubscriptBox[
StyleBox[\"k\",\nFontSlant->\"Italic\"], \(X\)]\)\[Psi](ln 2)(1-\!\(\*SubscriptBox[
StyleBox[\"k\",\nFontSlant->\"Italic\"], \(Y\)]\)\!\(\*
StyleBox[\"h\",\nFontSlant->\"Italic\"]\))(\!\(\*SubscriptBox[
StyleBox[\"R\",\nFontSlant->\"Italic\"], \(U\)]\)-\!\(\*SubscriptBox[
StyleBox[\"R\",\nFontSlant->\"Italic\"], \(Y\)]\))/(20\!\(\*SubscriptBox[
StyleBox[\"R\",\nFontSlant->\"Italic\"], \(U\)]\)\!\(\*SubscriptBox[
StyleBox[\"R\",\nFontSlant->\"Italic\"], \(Y\)]\))", FontFamily -> "Arial",
    14, Black],
   Style["Evolutionary Substitution assured", FontFamily -> "Arial", 14,
    Black]}, LegendLayout -> (Grid[#, Spacings -> {.5, .5},
     Alignment -> Left] &)], Below],
 PlotStyle -> {Directive[RGBColor[{0.810525, 0.852034, 0.912939}]],
   Directive[RGBColor[{0.964217, 0.883312, 0.742615}]],
   Directive[RGBColor[{0.868054, 0.907471, 0.758465}]]}]
```
