# Supplementary material for: Genesis of ectosymbiotic features based on commensalistic syntrophy
Source: Sci Rep. 2024 Jan 16;14:1366. doi: 10.1038/s41598-023-47211-8 (PMC10791676; doi:10.1038/s41598-023-47211-8)
Supplement: Supplementary file 2 — Supplementary Information 2. [file 41598_2023_47211_MOESM2_ESM.pdf]

Genesis of ectosymbiotic features based on commensalistic syntrophy

Nandakishor Krishnan [1,2,*], Villő Csiszár [3], Tamás F. Móri [4], József Garay [1]

[1] HUN-REN Centre for Ecological Research, Institute of Evolution, Konkoly-Thege M. út 29-33, Budapest 1121, Hungary

[2] Doctoral School of Biology, Institute of Biology, Eötvös Loránd University, Pázmány Péter sétány 1/C, Budapest 1117, Hungary

[3] Department of Probability Theory and Statistics, Eötvös Loránd University, Pázmány Péter sétány 1/C, Budapest 1117, Hungary

[4] HUN-REN Alfréd Rényi Institute of Mathematics, Reáltanoda u. 13-15, Budapest 1053, Hungary

email: nandakishor.kris@gmail.com (* Corresponding author)

SUPPLEMENTARY INFORMATION

SI (1) Equilibria and local stability analysis of the resident system

Based on the description of our resident model, we have the following three-dimensional dynamical system:

$$\dot{x} = x\left(\frac{k_X\psi - C_X}{R_X}\ln 2 - \frac{1}{P_X} - a_1 x\right)$$

$$\dot{y} = y\left(\frac{k_Y\frac{w}{V} - C_Y}{R_Y}\ln 2 - \frac{1}{P_Y} - a_2 y\right)$$

$$\dot{w} = k_X\psi x - k_Y\frac{w}{V}y$$

where all the parameters $k_X \in (0,1)$, $\psi \in \mathbb{R}_+$, $C_X \in (0,5)$, $R_X \in (0,5)$, $P_X > 1$, $a_1 \in \mathbb{R}_+$, $a_2 \in \mathbb{R}_+$, $k_Y \in (0,1)$, $C_Y \in (0,2)$, $R_Y \in (0,1)$, $P_Y > 1$ and $V \in \mathbb{R}_+$ are strictly positive real and are defined as in Table 1 of the main text.

For simplicity, let us first redefine the system as following:

$$\dot{x} = x(\alpha - a_1 x) \quad (1)$$

$$\dot{y} = y(\eta w - \rho - a_2 y) \quad (2)$$

$$\dot{w} = \phi x - \xi w y \quad (3)$$

where all the parameters $\alpha, \eta, \rho, \phi, \xi$ are strictly positive and defined as follows,

$$\alpha = \alpha_X = \frac{k_X\psi - C_X}{R_X}\ln 2 - \frac{1}{P_X}$$

$$\eta = \frac{k_Y}{VR_Y}\ln 2, \qquad \rho = \frac{C_Y}{R_Y}\ln 2 + \frac{1}{P_Y}$$

$$\phi = k_X\psi, \qquad \xi = \frac{k_Y}{V}$$

Next, we investigate the dynamics of the system in $\mathbb{R}^3_{\geq 0}$. The system (1) – (3) has two biologically feasible equilibria: $E_0(0,0,0)$ and $E_R(x^*, y^*, w^*)$ such that,

$$x^* = \frac{\alpha}{a_1}$$

$$y^* = \frac{\sqrt{\rho^2 + \frac{4a_2\alpha\eta\phi}{a_1\xi}} - \rho}{2a_2}$$

$$w^* = \frac{\sqrt{\rho^2 + \frac{4a_2\alpha\eta\phi}{a_1\xi}} + \rho}{2\eta}$$

Clearly, both equilibria $E_0(0,0,0)$ and $E_R(x^*, y^*, w^*)$ exist always. Now, we investigate the stability of the resident system at both equilibria using linearization.

The Jacobian matrix $J_{(x,y,w)}$ of the dynamical system at any arbitrary point (x, y, w) is given by,

$$J_{(x,y,w)} = \begin{pmatrix} \alpha - 2a_1x & 0 & 0 \\ 0 & \eta w - \rho - 2a_2y & \eta y \\ \phi & -\xi w & -\xi y \end{pmatrix}$$

For the local asymptotic stability of the equilibrium point, all eigenvalues of the Jacobian matrix must be negative. $E_0(0,0,0)$ is unstable as one eigenvalue of $J_{(0,0,0)}$ is positive.

Theorem 1.1 (Local stability of the positive equilibrium in the resident system) *The interior equilibrium* $E_R(x^*, y^*, w^*)$ *of the resident system (1) – (3) is locally asymptotically stable unconditionally.*

Proof:

Jacobian matrix at (x^*, y^*, w^*),

$$J_{(x^*,y^*,w^*)} = \begin{pmatrix} \alpha - 2a_1x^* & 0 & 0 \\ 0 & \eta w^* - \rho - 2a_2y^* & \eta y^* \\ \phi & -\xi w^* & -\xi y^* \end{pmatrix}$$

According to Routh Hurwitz criterion, the eigenvalues of the Jacobian matrix are negative for the following conditions:

1) $\operatorname{tr} J_{(x^*,y^*,w^*)} < 0$,
2) $\det J_{(x^*,y^*,w^*)} < 0$,
3) $\Lambda = \det\begin{pmatrix} J_{11} & 0 \\ 0 & J_{22} \end{pmatrix} + \det\begin{pmatrix} J_{11} & 0 \\ J_{31} & J_{33} \end{pmatrix} + \det\begin{pmatrix} J_{22} & J_{23} \\ J_{32} & J_{33} \end{pmatrix} > 0$, and
4) $\operatorname{tr} J_{(x^*,y^*,w^*)}\Lambda < \det J_{(x^*,y^*,w^*)}$

$$J_{(x^*,y^*,w^*)} = \begin{pmatrix} \alpha - 2a_1x^* & 0 & 0 \\ 0 & \eta w^* - \rho - 2a_2y^* & \eta y^* \\ \phi & -\xi w^* & -\xi y^* \end{pmatrix} := \begin{pmatrix} J_{11} & 0 & 0 \\ 0 & J_{22} & J_{23} \\ J_{31} & J_{32} & J_{33} \end{pmatrix}$$

$$J_{11} = \alpha - 2a_1x^* = -\alpha < 0$$

$$J_{33} = -\xi y^* < 0$$

$$J_{22} = \eta w^* - \rho - 2a_2 y^* = \eta \frac{\sqrt{\rho^2 + \frac{4a_2\alpha\eta\phi}{a_1\xi}} + \rho}{2\eta} - \rho - 2a_2 \frac{\sqrt{\rho^2 + \frac{4a_2\alpha\eta\phi}{a_1\xi}} - \rho}{2a_2}$$

$$= \frac{\sqrt{\rho^2 + \frac{4a_2\alpha\eta\phi}{a_1\xi}} + \rho}{2} - \rho - \sqrt{\rho^2 + \frac{4a_2\alpha\eta\phi}{a_1\xi}} + \rho < 0$$

All diagonal elements of $J_{(x^*,y^*,w^*)}$ are negative and hence $\mathrm{tr}\, J_{(x^*,y^*,w^*)} < 0$. Also, since $J_{23} = \eta y^* > 0$ and $J_{32} = -\xi w^* < 0$, we get $J_{22}J_{33} - J_{23}J_{32} > 0$. Therefore, $\det J_{(x^*,y^*,w^*)} = J_{11}(J_{22}J_{33} - J_{23}J_{32}) < 0$ and

$\Lambda = \det\begin{pmatrix} J_{11} & 0 \\ 0 & J_{22} \end{pmatrix} + \det\begin{pmatrix} J_{11} & 0 \\ J_{31} & J_{33} \end{pmatrix} + \det\begin{pmatrix} J_{22} & J_{23} \\ J_{32} & J_{33} \end{pmatrix} > 0$. Similarly, we can prove that,

$\mathrm{tr}\, J_{(x^*,y^*,w^*)}\Lambda - \det J_{(x^*,y^*,w^*)} = (J_{11} + J_{22} + J_{33})(J_{11}J_{22} + J_{11}J_{33} + J_{22}J_{33} - J_{23}J_{32}) - J_{11}(J_{22}J_{33} - J_{23}J_{32}) < 0$ (as all the individual terms in the expansion are negative).

Alternative method:

The eigenvalues of $J_{(x^*,y^*,w^*)}$ are,

$$J_{11}, \frac{1}{2}\left(J_{22} + J_{33} - \sqrt{J_{22}{}^2 + 4J_{23}J_{32} - 2J_{22}J_{33} + J_{33}{}^2}\right), \frac{1}{2}\left(J_{22} + J_{33} + \sqrt{J_{22}{}^2 + 4J_{23}J_{32} - 2J_{22}J_{33} + J_{33}{}^2}\right)$$

All eigenvalues are negative as $J_{11}, J_{22}, J_{33} < 0$ and $J_{23}J_{32} < 0$.

We can observe that all the eigenvalues of the linearized system at (x^*, y^*, w^*) are real and negative. Therefore, $E_R(x^*, y^*, w^*)$ is locally asymptotically stable always.

SI (2) Equilibria and local stability analysis of the resident-mutant coevolutionary system

Based on the description of our resident-mutant coevolutionary model, we have the following five-dimensional dynamical system:

$$\dot{x} = x\left(\frac{k_X\psi - C_X}{R_X}\ln 2 - \frac{1}{P_X} - a_1(x+z)\right) - \frac{\beta x u}{h}$$

$$\dot{y} = y\left(\frac{k_Y\frac{w}{V} - C_Y}{R_Y}\ln 2 - \frac{1}{P_Y} - a_2(y+u)\right)$$

$$\dot{z} = z\left(\frac{k_X\psi - C_X}{R_X}\ln 2 - \frac{1}{P_X} - a_1(x+z)\right) + \frac{\beta x u}{h}$$

$$\dot{u} = u\left(\frac{k_Y\frac{w}{V} - C_U}{R_U}\ln 2 - \frac{1}{P_Y} - a_2(y+u)\right) - \beta x u + \chi z$$

$$\dot{w} = k_X\psi x + (1 - k_Y h)k_X\psi z - k_Y\frac{w}{V}(y+u)$$

where all the parameters $k_X \in (0,1)$, $\psi \in \mathbb{R}_+$, $C_X \in (0,5)$, $R_X \in (0,5)$, $P_X > 1$, $a_1 \in \mathbb{R}_+$, $a_2 \in \mathbb{R}_+$, $k_Y \in (0,1)$, $C_Y \in (0,2)$, $R_Y \in (0,1)$, $C_U \in (0,2)$, $R_U \in (0,1)$, $P_Y > 1$, $\beta \in (0,1)$, $h \in \mathbb{R}_+$, $\chi \in \mathbb{R}_+$ and $V \in \mathbb{R}_+$ are strictly positive real and are defined as in Table 1 of the main text.

Let us first redefine the system as following:

$$\dot{x} = x(\alpha - a_1 x - a_1 z - (\beta/h)u) \quad (4)$$

$$\dot{y} = y(\eta w - \rho - a_2 y - a_2 u) \quad (5)$$

$$\dot{z} = z(\alpha - a_1 x - a_1 z) + (\beta/h)xu \quad (6)$$

$$\dot{u} = u(\mu w - \sigma - a_2 y - a_2 u - \beta x) + \chi z \quad (7)$$

$$\dot{w} = \phi x + \kappa\phi z - \xi w y - \xi w u \quad (8)$$

where all the parameters $\alpha, \eta, \rho, \phi, \xi, \mu, \sigma, \kappa$ are strictly positive and are defined as follows:

$$\alpha = \frac{k_{\mathrm{X}}\psi - C_{\mathrm{X}}}{R_{\mathrm{X}}}\ln 2 - \frac{1}{P_{\mathrm{X}}}$$

$$\eta = \frac{k_{\mathrm{Y}}}{VR_{\mathrm{Y}}}\ln 2, \qquad \rho = \frac{C_{\mathrm{Y}}}{R_{\mathrm{Y}}}\ln 2 + \frac{1}{P_{\mathrm{Y}}}$$

$$\phi = k_{\mathrm{X}}\psi, \qquad \xi = \frac{k_{\mathrm{Y}}}{V}$$

$$\mu = \frac{k_{\mathrm{Y}}}{VR_{\mathrm{U}}}\ln 2, \qquad \sigma = \frac{C_{\mathrm{U}}}{R_{\mathrm{U}}}\ln 2 + \frac{1}{P_{\mathrm{Y}}}$$

$$\kappa = 1 - k_{\mathrm{Y}}h$$

Next, we investigate the stability of the coevolutionary system (4) – (8) in $\mathbb{R}^5_{\geq 0}$. The system has four biologically feasible equilibria: $E_0(0,0,0,0,0)$, $E_1(x^*, y^*, 0, 0, w^*)$, $E_2(0, 0, z^+, u^+, w^+)$ and $E_3(0, \tilde{y}, \tilde{z}, \tilde{u}, \tilde{w})$. Positive-valued interior equilibria (x, y, z, u, w) such that $x, y, z, u, w \in \mathbb{R}_+$ do not exist for the above mentioned 5-D system.

Existence and Local Stability of $E_1(x^*, y^*, 0, 0, w^*)$

Consider the equilibrium point $(x^*, y^*, 0, 0, w^*)$ of the redefined system (4) – (8), where,

$$x^* = \frac{\alpha}{a_1}$$

$$y^* = \frac{\sqrt{\rho^2 + \frac{4a_2\alpha\eta\phi}{a_1\xi}} - \rho}{2a_2}$$

$$w^* = \frac{\sqrt{\rho^2 + \frac{4a_2\alpha\eta\phi}{a_1\xi}} + \rho}{2\eta}$$

As mentioned before, the resident equilibrium point of the system exists always unconditionally. We now conduct the stability analysis of this equilibrium point using linearization.

Theorem 2.1 (Local stability of $E_0(0,0,0,0,0)$ and $E_1(x^*, y^*, 0, 0, w^*)$)

The equilibrium points $E_0(0,0,0,0,0)$ and $E_1(x^*, y^*, 0, 0, w^*)$ of the coevolutionary system (4) – (8) are always unstable.

Proof:

The Jacobian matrix $J_{(x,y,z,u,w)}$ of the dynamical system at any arbitrary point (x, y, z, u, w) is given by,

$$J_{(x,y,z,u,w)} = \begin{pmatrix} \alpha - 2a_1x - a_1z - (\beta/h)u & 0 & -a_1x & -(\beta/h)x & 0 \\ 0 & \eta w - \rho - 2a_2y - a_2u & 0 & -a_2y & \eta y \\ -a_1z + (\beta/h)u & 0 & \alpha - a_1x - 2a_1z & (\beta/h)x & 0 \\ -\beta u & -a_2u & \chi & \mu w - \sigma - a_2y - 2a_2u - \beta x & \mu u \\ \phi & -\xi w & \phi\kappa & -\xi w & -\xi y - \xi u \end{pmatrix}$$

$E_0(0,0,0,0,0)$ is unstable as one eigenvalue of $J_{(0,0,0,0,0)}$ is positive.

Jacobian matrix at $(x^*, y^*, 0, 0, w^*)$:

$$J_{(x^*,y^*,0,0,w^*)} = \begin{pmatrix} \alpha - 2a_1x^* & 0 & -a_1x^* & -(\beta/h)x^* & 0 \\ 0 & \eta w^* - \rho - 2a_2y^* & 0 & -a_2y^* & \eta y^* \\ 0 & 0 & \alpha - a_1x^* & (\beta/h)x^* & 0 \\ 0 & 0 & \chi & \mu w^* - \sigma - a_2y^* - \beta x^* & 0 \\ \phi & -\xi w^* & \phi\kappa & -\xi w^* & -\xi y^* \end{pmatrix}$$

$$:= \begin{pmatrix} J_{11} & 0 & J_{13} & J_{14} & 0 \\ 0 & J_{22} & 0 & J_{24} & J_{25} \\ 0 & 0 & 0 & J_{34} & 0 \\ 0 & 0 & J_{43} & J_{44} & 0 \\ J_{51} & J_{52} & J_{53} & J_{54} & J_{55} \end{pmatrix}$$

The eigenvalues are:

1) J_{11},
2) $\frac{1}{2}\left(J_{44} - \sqrt{4J_{34}J_{43} + J_{44}^2}\right)$,
3) $\frac{1}{2}\left(J_{44} + \sqrt{4J_{34}J_{43} + J_{44}^2}\right)$,
4) $\frac{1}{2}\left(J_{22} + J_{55} - \sqrt{J_{22}^2 + 4J_{25}J_{52} - 2J_{22}J_{55} + J_{55}^2}\right)$, and
5) $\frac{1}{2}\left(J_{22} + J_{55} + \sqrt{J_{22}^2 + 4J_{25}J_{52} - 2J_{22}J_{55} + J_{55}^2}\right)$

We can observe that the third eigenvalue is never negative since $J_{34}J_{43} > 0$. Therefore $E_1(x^*, y^*, 0, 0, w^*)$ is unstable.

Existence and Local Stability of $E_M(z^+, u^+, w^+)$ and $E_2(0, 0, z^+, u^+, w^+)$

Now consider the equilibrium point $(0, 0, z^+, u^+, w^+)$ of the system (4) – (8) such that,

$$z^+ = \frac{\alpha}{a_1}$$

$$u^+ = \frac{\sqrt{\sigma^2 + \frac{4a_2\alpha(\mu\phi\kappa + \chi\xi)}{a_1\xi}} - \sigma}{2a_2}$$

$$w^+ = \frac{2\alpha\phi\kappa a_2}{a_1\xi\left(\sqrt{\sigma^2 + \frac{4a_2\alpha(\mu\phi\kappa + \chi\xi)}{a_1\xi}} - \sigma\right)} = \frac{\phi\kappa\left(\sqrt{\sigma^2 + \frac{4a_2\alpha(\mu\phi\kappa + \chi\xi)}{a_1\xi}} + \sigma\right)}{2(\mu\phi\kappa + \chi\xi)}$$

The mutant rest point of the system exists always. We first conduct the stability analysis of the mutant-only equilibrium point in three-dimension.

Consider the following system with only the mutant phenotype of the symbiont,

$$\dot{z} = z(\alpha - a_1 z) \tag{9}$$

$$\dot{u} = u(\mu w - \sigma - a_2 u) + \chi z \tag{10}$$

$$\dot{w} = \kappa\phi z - \xi w u \tag{11}$$

where all parameters are defined as in the five-dimensional coevolutionary system (4) – (8).

Jacobian matrix at (z^+, u^+, w^+),

$$J_{(z^+,u^+,w^+)} = \begin{pmatrix} \alpha - 2a_1 z^+ & 0 & 0 \\ \chi & \mu w^+ - \sigma - 2a_2 u^+ & \mu u^+ \\ \kappa\phi & -\xi w^+ & -\xi u^+ \end{pmatrix} := \begin{pmatrix} J_{11} & 0 & 0 \\ J_{21} & J_{22} & J_{23} \\ J_{31} & J_{32} & J_{33} \end{pmatrix}$$

$$J_{11} = \alpha - 2a_1 z^+ = -\alpha < 0$$

$$J_{33} = -\xi u^+ < 0$$

$$J_{22} = \mu w^+ - \sigma - 2a_2 u^+ = \mu \frac{\phi\kappa\left(\sqrt{\sigma^2 + \frac{4a_2\alpha(\mu\phi\kappa + \chi\xi)}{a_1\xi}} + \sigma\right)}{2(\mu\phi\kappa + \chi\xi)} - \sigma - 2a_2 \frac{\sqrt{\sigma^2 + \frac{4a_2\alpha(\mu\phi\kappa + \chi\xi)}{a_1\xi}} - \sigma}{2a_2}$$

$$= \frac{\mu\phi\kappa\left(\sqrt{\sigma^2 + \frac{4a_2\alpha(\mu\phi\kappa + \chi\xi)}{a_1\xi}} + \sigma\right) - 2(\mu\phi\kappa + \chi\xi)\sqrt{\sigma^2 + \frac{4a_2\alpha(\mu\phi\kappa + \chi\xi)}{a_1\xi}}}{2(\mu\phi\kappa + \chi\xi)} < 0$$

The eigenvalues of $J_{(z^+,u^+,w^+)}$ are:

$$J_{11}, \frac{1}{2}\left(J_{22} + J_{33} - \sqrt{J_{22}^2 + 4J_{23}J_{32} - 2J_{22}J_{33} + J_{33}^2}\right), \frac{1}{2}\left(J_{22} + J_{33} + \sqrt{J_{22}^2 + 4J_{23}J_{32} - 2J_{22}J_{33} + J_{33}^2}\right)$$

All eigenvalues are real and negative as $J_{11}, J_{22}, J_{33} < 0$ and $J_{23}J_{32} < 0$. Therefore, the interior equilibrium $E_M(z^+, u^+, w^+)$ of the mutant-only system (9) – (11) is locally asymptotically stable always.

We now conduct the stability analysis of the mutant equilibrium point of the five-dimensional resident-mutant system (4) – (8).

Theorem 2.2 (Local stability of $E_2(0, 0, z^+, u^+, w^+)$) *The equilibrium $E_2(0, 0, z^+, u^+, w^+)$ of the system (4) – (8) is locally asymptotically stable if:*

1) $\frac{c_Y}{R_Y} > \frac{c_U}{R_U}$ and
2) $\chi > \frac{k_X \psi (\ln 2)(1 - k_Y h)(R_U - R_Y)}{R_U R_Y}$

Proof:

Jacobian matrix at $(0, 0, z^+, u^+, w^+)$:

$$J_{(0,0,z^+,u^+,w^+)} = \begin{pmatrix} \alpha - a_1 z^+ - (\beta/h)u^+ & 0 & 0 & 0 & 0 \\ 0 & \eta w^+ - \rho - a_2 u^+ & 0 & 0 & 0 \\ -a_1 z^+ + (\beta/h)u^+ & 0 & \alpha - 2a_1 z^+ & 0 & 0 \\ -\beta u^+ & -a_2 u^+ & \chi & \mu w^+ - \sigma - 2a_2 u^+ & \mu u^+ \\ \phi & -\xi w^+ & \phi\kappa & -\xi w^+ & -\xi u^+ \end{pmatrix}$$

The characteristic polynomial corresponding to the Jacobian matrix (if λ is an eigenvalue) is,

$$(\alpha - a_1 z^+ - (\beta/h)u^+ - \lambda)(\eta w^+ - \rho - a_2 u^+ - \lambda)(\alpha - 2a_1 z^+ - \lambda)[\lambda^2 + \lambda(2a_2 u^+ + \xi u^+ + \sigma - \mu w^+) + (\sigma\xi u^+ + 2a_2\xi {u^+}^2)] = 0$$

All eigenvalues are negative if:

1) $\alpha - a_1 z^+ - (\beta/h)u^+ < 0$
2) $\alpha - 2a_1 z^+ < 0$
3) $\sigma\xi u^+ + 2a_2\xi {u^+}^2 > 0$
4) $2a_2 u^+ + \xi u^+ + \sigma - \mu w^+ > 0$ and
5) $\eta w^+ - \rho - a_2 u^+ < 0$

Clearly, $\alpha - a_1 z^+ - (\beta/h)u^+ < 0$, $\alpha - 2a_1 z^+ < 0$ and $\sigma\xi u^+ + 2a_2\xi {u^+}^2 > 0$.

Proof of $2a_2 u^+ + \xi u^+ + \sigma - \mu w^+ > 0$:

$$2a_2 u^+ + \sigma - \mu w^+ = \sqrt{\sigma^2 + \frac{4a_2\alpha(\mu\phi\kappa + \chi\xi)}{a_1\xi}} - \frac{2\alpha\phi\kappa\mu a_2}{a_1\xi\left(\sqrt{\sigma^2 + \frac{4a_2\alpha(\mu\phi\kappa + \chi\xi)}{a_1\xi}} - \sigma\right)}$$

On simplifying to a fraction, denominator is positive.

Numerator is equal to,

$$a_1\xi\left(\sqrt{\sigma^2 + \frac{4a_2\alpha(\mu\phi\kappa + \chi\xi)}{a_1\xi}} - \sigma\right)\sqrt{\sigma^2 + \frac{4a_2\alpha(\mu\phi\kappa + \chi\xi)}{a_1\xi}} - 2\alpha\phi\kappa\mu a_2$$

$$= a_1\xi\left(\sigma^2 + \frac{4a_2\alpha(\mu\phi\kappa + \chi\xi)}{a_1\xi} - \sigma\sqrt{\sigma^2 + \frac{4a_2\alpha(\mu\phi\kappa + \chi\xi)}{a_1\xi}}\right) - 2\alpha\phi\kappa\mu a_2$$

$$= \left(a_1\xi\sigma^2 + 4a_2\alpha(\mu\phi\kappa + \chi\xi) - a_1\xi\sigma\sqrt{\sigma^2 + \frac{4a_2\alpha(\mu\phi\kappa + \chi\xi)}{a_1\xi}}\right) - 2\alpha\phi\kappa\mu a_2$$

$$= \left(a_1\xi\sigma^2 - a_1\xi\sigma\sqrt{\sigma^2 + \frac{4a_2\alpha(\mu\phi\kappa + \chi\xi)}{a_1\xi}}\right) + 4a_2\alpha\chi\xi + 2\alpha\phi\kappa\mu a_2$$

$$= a_1\xi\sigma\left(\sigma - \sqrt{\sigma^2 + \frac{4a_2\alpha(\mu\phi\kappa + \chi\xi)}{a_1\xi}}\right) + 2a_2\alpha(\mu\phi\kappa + 2\chi\xi)$$

$$= a_1\xi\sigma\left(\sigma - \sqrt{\sigma^2 + \frac{4a_2\alpha(\mu\phi\kappa + \chi\xi)}{a_1\xi}} + \frac{2a_2\alpha(\mu\phi\kappa + 2\chi\xi)}{a_1\xi\sigma}\right) > 0$$

Thus, numerator is positive. Therefore, $2a_2 u^+ + \sigma - \mu w^+ > 0$, which implies,

$$2a_2 u^+ + \xi u^+ + \sigma - \mu w^+ > 0$$

Therefore, the only sufficient condition required for the local asymptotic stability of $(0, 0, z^+, u^+, w^+)$ is,

$$\eta w^+ - \rho - a_2 u^+ < 0$$

Proof of $\eta w^+ - \rho - a_2 u^+ < 0$ if $\rho > \sigma$ and $\chi\xi + (\mu - \eta)\phi\kappa > 0$:

$$\eta w^+ - a_2 u^+$$

$$= \frac{-1}{2}\left(-\sigma + \sqrt{\sigma^2 + \frac{4a_2\alpha(\mu\phi\kappa + \chi\xi)}{a_1\xi}}\right) + \frac{2\alpha\eta\phi\kappa a_2}{a_1\xi\left(-\sigma + \sqrt{\sigma^2 + \frac{4a_2\alpha(\mu\phi\kappa + \chi\xi)}{a_1\xi}}\right)}$$

$$= \frac{-a_1\xi\sigma^{\,2} - 2\alpha a_2(\mu\phi\kappa + \chi\xi) + a_1\xi\sigma\sqrt{\sigma^2 + \frac{4a_2\alpha(\mu\phi\kappa + \chi\xi)}{a_1\xi}} + 2\alpha\eta\phi\kappa a_2}{a_1\xi\left(-\sigma + \sqrt{\sigma^2 + \frac{4a_2\alpha(\mu\phi\kappa + \chi\xi)}{a_1\xi}}\right)}$$

$$= \frac{-a_1\xi\sigma\left(\sigma - \sqrt{\sigma^2 + \frac{4a_2\alpha(\mu\phi\kappa + \chi\xi)}{a_1\xi}}\right) - 2\alpha a_2(\mu\phi\kappa + \chi\xi) + 2\alpha\eta\phi\kappa a_2}{a_1\xi\left(-\sigma + \sqrt{\sigma^2 + \frac{4a_2\alpha(\mu\phi\kappa + \chi\xi)}{a_1\xi}}\right)}$$

$$= \sigma - \frac{2\alpha a_2(\mu\phi\kappa + \chi\xi - \eta\phi\kappa)}{a_1\xi\left(-\sigma + \sqrt{\sigma^2 + \frac{4a_2\alpha(\mu\phi\kappa + \chi\xi)}{a_1\xi}}\right)}$$

Thus, sufficient conditions for $\eta w^+ - \rho - a_2 u^+ < 0$ is $\rho > \sigma$ and $\chi\xi + (\mu - \eta)\phi\kappa > 0$.

Recall that $\rho = \frac{C_\mathrm{Y}}{R_\mathrm{Y}}\ln 2 + \frac{1}{P_\mathrm{Y}}$ and $\sigma = \frac{C_\mathrm{U}}{R_\mathrm{U}}\ln 2 + \frac{1}{P_\mathrm{Y}}$.

$$\rho > \sigma \Rightarrow \frac{C_\mathrm{Y}}{R_\mathrm{Y}}\ln 2 + \frac{1}{P_\mathrm{Y}} > \frac{C_\mathrm{U}}{R_\mathrm{U}}\ln 2 + \frac{1}{P_\mathrm{Y}}$$

$$\Rightarrow \frac{C_\mathrm{Y}}{R_\mathrm{Y}} > \frac{C_\mathrm{U}}{R_\mathrm{U}}$$

$$\Rightarrow C_\mathrm{Y}R_\mathrm{U} > C_\mathrm{U}R_\mathrm{Y}$$

Recall that $\mu = \frac{k_\mathrm{Y}}{VR_\mathrm{U}}\ln 2$, $\eta = \frac{k_\mathrm{Y}}{VR_\mathrm{Y}}\ln 2$, $\phi = k_\mathrm{X}\psi$, $\xi = \frac{k_\mathrm{Y}}{V}$, $\kappa = 1 - k_\mathrm{Y}h$.

Therefore,

$$\chi\xi + (\mu - \eta)\phi\kappa > 0 \Rightarrow \frac{k_\mathrm{X}\psi(\ln 2)(1 - k_\mathrm{Y}h)k_\mathrm{Y}}{V}\left(\frac{1}{R_\mathrm{U}} - \frac{1}{R_\mathrm{Y}}\right) + \chi\frac{k_\mathrm{Y}}{V} > 0$$

Sufficient conditions for the stability of $E_2(0, 0, z^+, u^+, w^+)$ (i.e., evolutionary substitution of a resident phenotype by the mutant) are:

1) $\frac{C_\mathrm{Y}}{R_\mathrm{Y}} > \frac{C_\mathrm{U}}{R_\mathrm{U}}$
2) $\chi > \frac{k_\mathrm{X}\psi(\ln 2)(1 - k_\mathrm{Y}h)(R_\mathrm{U} - R_\mathrm{Y})}{R_\mathrm{U}R_\mathrm{Y}}$

Existence of $E_3(0, \tilde{y}, \tilde{z}, \tilde{u}, \tilde{w})$

If the above-mentioned sufficient conditions hold, this rules out the existence of the rest point $E_3(0, \tilde{y}, \tilde{z}, \tilde{u}, \tilde{w})$. If these sufficient conditions for stability of $(0, 0, z^+, u^+, w^+)$ are not satisfied, then $(0, \tilde{y}, \tilde{z}, \tilde{u}, \tilde{w})$ can also exist additional to E_0, E_1 and E_2 under some conditions. Biologically, stability of E_3 implies polymorphism as the resident and mutant phenotypes of one species coexist.

The equilibrium point $E_3(0, \tilde{y}, \tilde{z}, \tilde{u}, \tilde{w})$ of the coevolutionary system (4) – (8) is obtained as,

$$\tilde{y} = \Big(-{a_1}^{3/2}\xi^{3/2}\rho(\rho-\sigma)(\mu\rho-\eta\sigma) + a_1\xi(\rho-\sigma)(\mu\rho-\eta\sigma)\sqrt{a_1\xi\rho^2+4a_2\alpha\eta\kappa\phi} + a_2\alpha(\eta - \mu)\sqrt{a_1\xi\rho^2+4a_2\alpha\eta\kappa\phi}(-\eta\kappa\phi+\kappa\mu\phi+\xi\chi) + \sqrt{a_1}a_2\alpha\sqrt{\xi}(\eta^2\kappa\rho\phi - 2\eta\kappa\mu\rho\phi + \eta\xi(\rho-2\sigma)\chi + \mu\rho(\kappa\mu\phi+\xi\chi))\Big) / \big(2\sqrt{a_1}a_2\sqrt{\xi}(a_1\xi(\rho-\sigma)(\mu\rho-\eta\sigma) - a_2\alpha\kappa(\eta-\mu)^2\phi)\big)$$

$$\tilde{z} = \frac{\alpha}{a_1}$$

$$\tilde{u} = \frac{-\sqrt{a_1}\alpha\xi(\mu\rho+\eta(\rho-2\sigma))\chi + \alpha(-\eta+\mu)\sqrt{\xi}\sqrt{a_1\xi\rho^2+4a_2\alpha\eta\kappa\phi}\chi}{2\sqrt{a_1}(a_1\xi(\rho-\sigma)(\mu\rho-\eta\sigma) - a_2\alpha\kappa(\eta-\mu)^2\phi)}$$

$$\widetilde{w} = \frac{\sqrt{\rho^2 + \frac{4a_2\alpha\eta\kappa\phi}{a_1\xi}} + \rho}{2\eta}$$

In this paper, we do not analytically study the stability of the rest point $(0, \tilde{y}, \tilde{z}, \tilde{u}, \widetilde{w})$ due to its complexity and because we are only concerned about the possibility of mutant invasion and subsequent fixation of a mutant system (mutant substituting resident phenotype) with the "consortia." However, we did some numerical investigation of all possible equilibria.

SI (3) Numerical Analysis

This section deals with the numerical investigations of the model to substantiate the analytical results. Let us first consider the following resident system:

$$\dot{x} = x(\alpha - a_1 x)$$

$$\dot{y} = y(\eta w - \rho - a_2 y)$$

$$\dot{w} = \phi x - \xi w y$$

where all the parameters $\alpha, \eta, \rho, \phi, \xi$ are strictly positive and defined as follows,

$$\alpha = \alpha_X = \frac{k_X\psi - C_X}{R_X}\ln 2 - \frac{1}{P_X}$$

$$\eta = \frac{k_Y}{VR_Y}\ln 2, \qquad \rho = \frac{C_Y}{R_Y}\ln 2 + \frac{1}{P_Y}$$

$$\phi = k_X\psi, \qquad \xi = \frac{k_Y}{V}$$

If $\psi = 400$, $a_1 = 3$, $a_2 = 2$, $k_X = 0.2$, $C_X = 4$, $R_X = 3$, $P_X = 10$, $k_Y = 0.1$, $R_Y = 0.3$, $C_Y = 1.2$, $P_Y = 3$, $\phi = 80$, $\xi = 0.01$, and $V = 10$.

The interior equilibrium point of the above system is then $E_R(x^*, y^*, w^*) = (5.82, 22.43, 2076)$.

Now consider the five-dimensional resident-mutant coevolutionary system.

If conditions for evolutionary substitution are satisfied, $\rho > \sigma$ and $\chi = 30$:

Consider the following system,

$$\dot{x} = x(\alpha - a_1 x - a_1 z - (\beta/h)u)$$

$$\dot{y} = y(\eta w - \rho - a_2 y - a_2 u)$$

$$\dot{z} = z(\alpha - a_1 x - a_1 z) + (\beta/h)xu$$

$$\dot{u} = u(\mu w - \sigma - a_2 y - a_2 u - \beta x) + \chi z$$

$$\dot{w} = \phi x + \kappa\phi z - \xi w y - \xi w u$$

where all the parameters $\alpha, \eta, \rho, \phi, \xi, \mu, \sigma, \kappa$ are strictly positive and are defined as follows:

$$\alpha = \frac{k_X\psi - C_X}{R_X}\ln 2 - \frac{1}{P_X}$$

$$\eta = \frac{k_Y}{VR_Y}\ln 2, \qquad \rho = \frac{C_Y}{R_Y}\ln 2 + \frac{1}{P_Y}$$

$$\phi = k_X\psi, \qquad \xi = \frac{k_Y}{V}$$

$$\mu = \frac{k_Y}{VR_U}\ln 2, \qquad \sigma = \frac{C_U}{R_U}\ln 2 + \frac{1}{P_Y}$$

$$\kappa = 1 - k_Y h$$

If $\beta = 0.8, h = 4, \chi = 30, R_U = 0.4, C_U = 1.3$, and rest of the parameters are same as in the previously mentioned resident system. Then, the equilibria of the coevolutionary system are $E_0(0,0,0,0,0)$, $E_1(x^*, y^*, 0, 0, w^*) = (5.82, 22.43, 0, 0, 2076)$, and $E_2(0, 0, z^+, u^+, w^+) = (0,\ 0,\ 5.82, 17.51, 1595)$.

If conditions for evolutionary substitution are not satisfied, $\rho < \sigma$ *and* $\chi = 5$:

Now if for the above five-dimensional coevolutionary system, $\beta = 0.8, h = 4, \chi = 5, R_U = 0.32, C_U = 1.3$, and rest of the parameters are same as in the previously mentioned resident system.

Then, the equilibria of the coevolutionary system are $E_0(0,0,0,0,0)$, $E_1(x^*, y^*, 0, 0, w^*) = (5.82, 22.43, 0, 0, 2076)$, $E_2(0,0,z^+, u^+, w^+) = (0, 0, 5.82, 17.04, 1639.7)$, and $E_3(0, \tilde{y}, \tilde{z}, \tilde{u}, \tilde{w}) = (0, 5.02, 5.82, 12.19, 1623.7)$

SI (4) Verification of probability of Evolutionary Substitution

Consider (as in SI (3)) $\psi = 400$, $k_X = 0.2$, $k_Y = 0.1$, $R_Y = 0.3$, $C_Y = 1.2, h = 4$, and $\chi^*(\text{ Scaled } \chi) = \chi/20$. The intersection area of the regions corresponding to the conditions of evolutionary substitution in Fig. S1 gives the minimum probability of evolutionary substitution.

Area of intersection (green) = Area of intersection of $C_Y < C_U < \frac{C_Y}{R_Y} R_U$ and $\chi^* > \frac{k_X\psi(\ln 2)(1 - k_Y h)(R_U - R_Y)}{20 R_U R_Y}$

$$= \text{Area of intersection of } 1.2 < y < 4x \text{ and } y > 5.55\left(\frac{x - 0.3}{x}\right)$$

$$= \int_{0.3}^{0.4387} 4x\,dx - \int_{0.3828}^{0.4387} 5.55\left(\frac{x - 0.3}{x}\right)dx - 1.2(0.3828 - 0.3) = 0.022$$

Therefore, the plot analysis verifies the non-zero probability of evolutionary substitution under arbitrary parameter values.

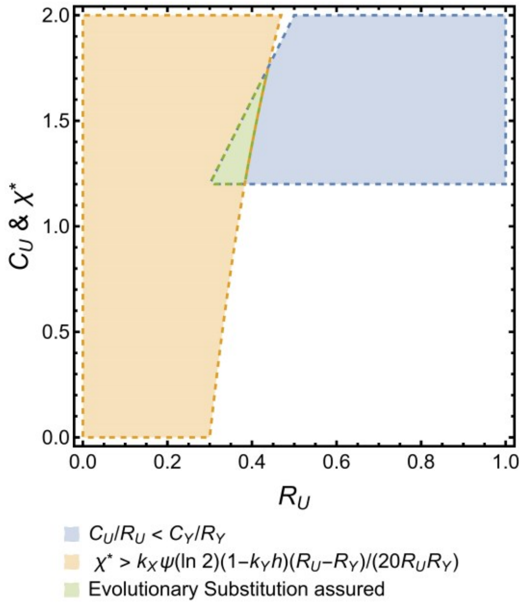

Figure S1. Region-plot with R_U on X-axis and C_U & scaled χ on Y-axis showing the sufficient conditions for evolutionary substitution of the resident phenotype by the mutant phenotype of the symbiont species. The values of all the other parameters are fixed as in SI (3). The figure is just a verification of the existence of plausible parameter values and to authenticate our analytical results. It is only a visualization of the two conditions (or inequalities) for evolutionary substitution. The figure only shows a small portion of the solution space, which guarantees evolutionary substitution under some parameter choices. A decrease in host consumption rate (k_X), decrease in food concentration (ψ), increase in symbiont consumption rate (k_Y), or increase in the number of ectosymbionts (h) will change the slope of one of the curves, opening more area of solution space. Note this does not give the entire solution space, as we provide the sufficient conditions that guarantee evolutionary substitution.

SI (5) Codes to reproduce analyses and figures

Numerical analysis and visualization of results were done in Mathematica 13.3 (Wolfram Research, Inc., Mathematica, Version 13.3, Champaign, IL, 2023, https://www.wolfram.com/mathematica). The Mathematica codes used are provided as a separate Supplementary file.
